# A perioperative medicine platform trial (PROMPT) evaluating interventions to reduce the risk of infection and other major complications after surgery: rationale and design

**DOI:** 10.1016/j.bjao.2026.100855

**Published:** 2026-08-28

**Authors:** Paul S. Myles, Trisha N. Peel, Steve Webb, Sophie K. Wallace, Wendy A. Brown, Tomas Corcoran, Tracey Bucknall, Tim Coulson, Daniel Frei, Brett Mitchell, Darshini Ayton, Alisa M. Higgins, Andrew Forbes, Ricki Spencer, Elizabeth G. Ryan

**Affiliations:** 1 Department of Anaesthesiology and Perioperative Medicine, Alfred Hospital and Monash University, Melbourne, Australia; 2 Department of Infectious Diseases, Alfred Hospital and Monash University, Melbourne, Australia; 3 School of Public Health and Preventive Medicine, Monash University, Melbourne, Australia; 4 Department of Surgery, Alfred Hospital and Monash University, Melbourne, Australia; 5 Department of Anaesthesia and Pain Medicine, Royal Perth Hospital and the School of Emergency Medicine, University of Western Australia, Perth, Australia; 6 Centre for Quality and Patient Safety Research, Institute for Health Transformation, School of Nursing and Midwifery, Deakin University, Geelong, Australia; 7 Department of Anaesthesiology and Pain Management, Wellington Hospital and Medical Research Institute of New Zealand, New Zealand; 8 School of Nursing and Health, Avondale University, New South Wales, Australia; 9 Perioperative Medicine Adaptive Platform Trial (PROMPT) Consumer Committee, Melbourne, Australia

**Keywords:** anaesthesia, Bayesian adaptive platform trial, complications, master protocol, surgery, surgical site infection

## Abstract

**Background:**

Platform trials are an extremely powerful, cost-effective innovation in clinical trial design and evidence-to-practice translation. As questions are answered progressively, additional new interventions, within the same or new domains, are substituted into the platform. This design is ideal for evaluation of interventions aiming to reduce surgical site infection, which remains the most common serious complication after surgery.

**Objective:**

To efficiently identify effective, ineffective, or harmful treatments to reduce the incidence of perioperative infections and other major complications after surgery.

**Study design:**

Perioperative medicine adaptive platform trial (PROMPT) is a multicentre, Bayesian adaptive platform trial designed to reach conclusions regarding superiority, efficacy, equivalence, inferiority, harm, or futility. Modules within the protocol include the master protocol, multiple domain-specific appendices, and a statistical appendix.

**Study population:**

Adult patients undergoing a broad range of surgical procedures in participating hospitals.

**Interventions:**

Specific interventions under study will be described in domain-specific appendices. The first intervention under study is liberal inspired oxygen concentrations during surgery.

**Outcomes:**

The primary outcome is the incidence of surgical site infection up to 30 days after index surgery; this will apply to all domains unless otherwise specified in a domain-specific appendix. Secondary outcomes include other health care–associated infections and major postoperative complications up to 30 days after surgery, quality of recovery (QoR-15) at 3 and 30 days after surgery, and days alive and at home up to 30 days after surgery (DAH_30_).

**Conclusions:**

PROMPT is designed to efficiently evaluate interventions aiming to reduce surgical site infection and other major complications after surgery.

**Clinical trial registration:**

NCT07186634.

**Protocol version number and date:**

1.1, December 4, 2025.

Surgery is being performed at the nexus of an ageing, highly comorbid population, an increasing breadth and complexity of surgical procedures, and increasingly resistant bacteria.

Surgical site infections (SSIs) are common, occurring in up to 15% of surgical procedures, and are often life-threatening and risk permanent disability. Potentially half of these infections are preventable.^1^^,^^2^ Approximately one-fifth of all infections acquired in hospitals are SSIs.^3^ In the recent World Health Organization Global Guidelines for the Prevention of SSI,^2^ none of the practice recommendations were based on high-quality evidence. Consequently, compliance with guidelines remains poor, and clinical practices and outcomes vary widely, with the incidence of SSIs remaining unacceptably high. Relevant and reliable evidence to reduce infections, and the associated poor outcomes and high health care costs, is urgently needed.

This article describes the rationale and design of the perioperative medicine adaptive platform trial (PROMPT). Adaptive platform trials are extremely powerful, cost-effective innovations in clinical trial design and evidence-to-practice translation.^4^, ^5^, ^6^, ^7^, ^8^ These have the proven potential to accelerate knowledge generation and identify effective, ineffective, or harmful treatments much faster and at lower cost compared with traditional designs. Translation into clinical practice and guidelines can be near-immediate.

## PROMPT objectives

To efficiently identify effective, ineffective, or harmful treatments to reduce the incidence of perioperative infections and other major postoperative complications. The initial domain to be evaluated in PROMPT will consist of three levels of inspired oxygen concentration (PROMPT-O_2_), as informed by our recent studies and editorials,^9^, ^10^, ^11^, ^12^, ^13^, ^14^, ^15^ and international guidelines.^2^^,^^16^^,^^17^ Future domains are planned to include glycaemic control and red cell transfusion thresholds in noncardiac surgery.

## Protocol structure

**Fig 1:**
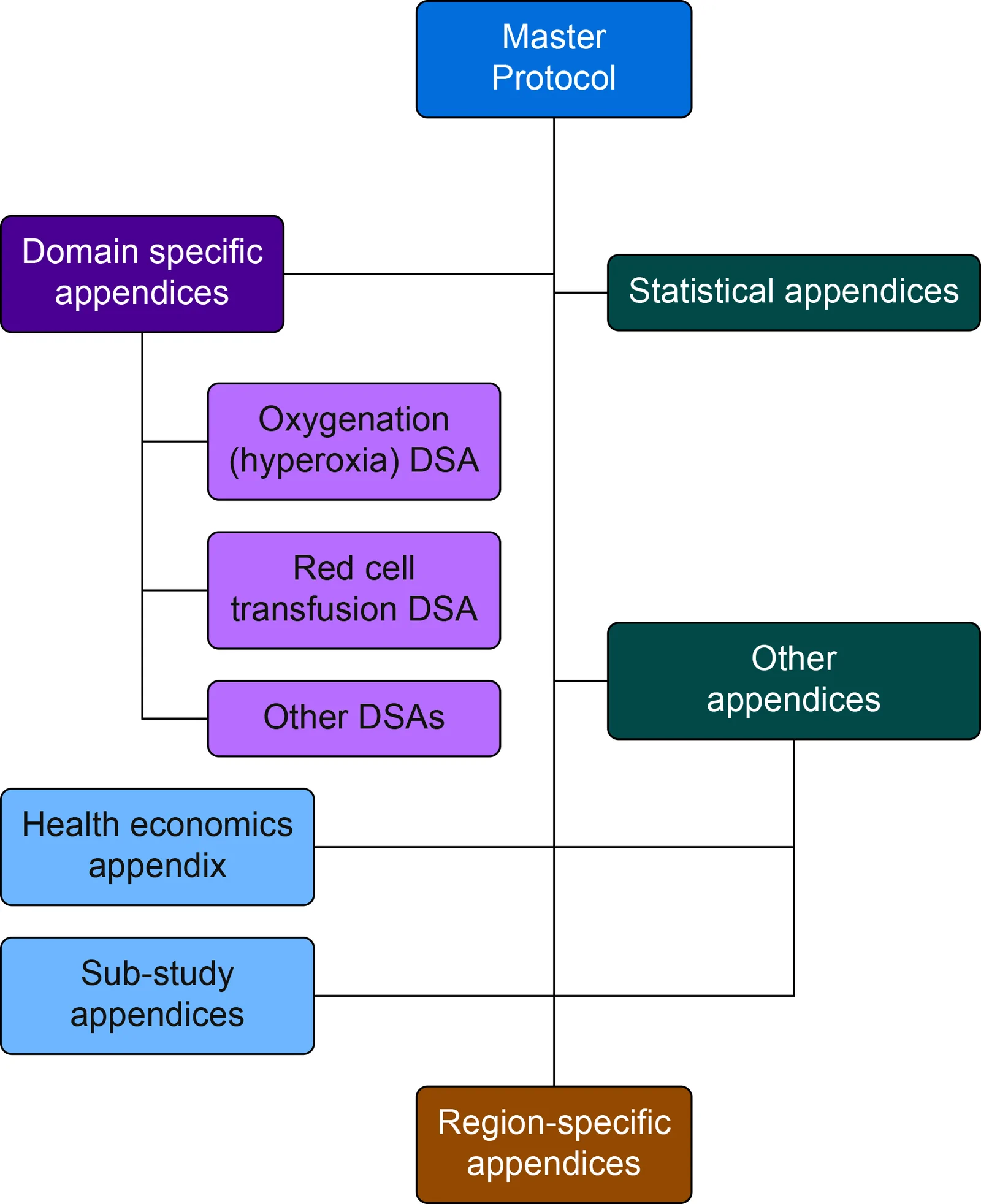
PROMPT protocol structure. DSA, domain-specific appendix; PROMPT, perioperative medicine adaptive platform trial.

The PROMPT protocol is presented in a modular format, reflecting the complexity of the adaptive platform trial structure. Modules within the protocol include the master protocol, multiple domain-specific appendices (DSAs), future region-specific appendices, a health economics appendix, and a statistical appendix. The structure of the PROMPT protocol is outlined in Fig. 1. A glossary of terms used across all PROMPT documents is presented in Supplementary Appendix S1.

The master protocol contains all information relevant to trial processes and applies to all regions and domains for the duration of the trial, in a hierarchical manner (Fig. 1). It contains the background and rationale for studying SSI as a problem of perioperative and public health interest, the rationale for using an adaptive platform trial structure, the trial design, platform-wide eligibility criteria, randomisation details, core trial outcomes, bias minimisation, principles of the statistical analyses, principles for performing the adaptive analyses, and adaptations to the trial that may occur, including early cessation of recruitment because of futility, superiority, or harm of an intervention within a domain.

Note that the platform-wide eligibility criteria, data collection, and secondary outcomes in the master protocol may be supplemented with those in the DSAs. Each domain generally has additional eligibility criteria, data points, and secondary outcome measures. The statistical appendix contains a detailed description of the statistical models used for estimating the effect of interventions (Supplementary Appendix S2), and also contains the simulation appendix that presents a range of simulation-based operating characteristics for each domain.

## Study design

**Fig 2:**
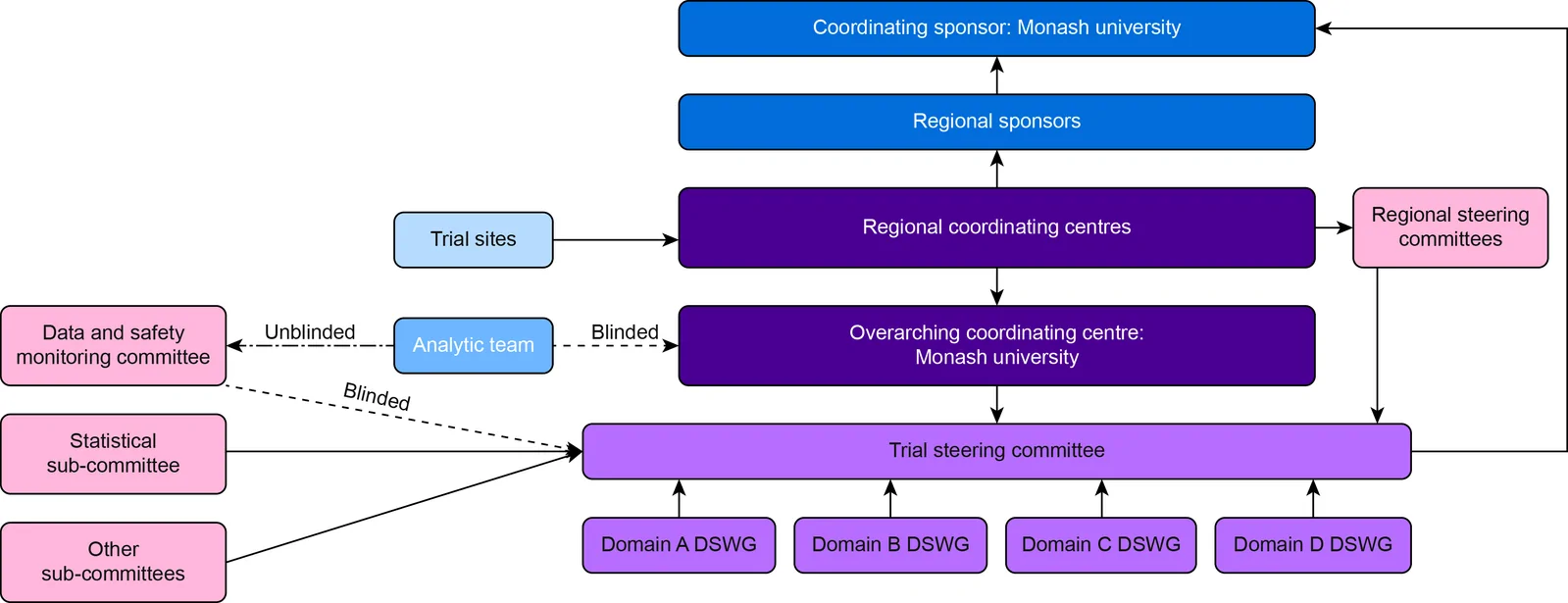
PROMPT trial structure. PROMPT, perioperative medicine adaptive platform trial.

PROMPT is a multicentre, embedded multifactorial platform trial that uses a Bayesian adaptive design framework to reach conclusions regarding the effectiveness of multiple therapeutic treatments to reduce the incidence of perioperative infections. The PROMPT trial flow is presented in Fig. 2. The core eligibility criteria are detailed in Table 1.

**Table 1:** PROMPT core eligibility criteria.

PROMPT core inclusion criteria
1. Adult patient (≥18 years of age at time of admission).
2. Scheduled to undergo a surgical procedure with a skin incision, expected duration of at least 2 hours, and a planned overnight hospital stay of at least 1 night (including, but not limited to gastrointestinal, neurosurgical, cardiac, orthopaedic, obstetric, or gynaecological surgery).
PROMPT core exclusion criteria
1. ASA physical status 5 (moribund, not expected to survive with or without an operation).
2. Poor spoken and/or written language comprehension.
3. Previous participation in PROMPT within the previous 30 days.

Each domain may have additional, domain-specific eligibility criteria. These additional eligibility criteria that are specific to a domain are provided in each DSA. Patients who fulfil the overall PROMPT eligibility criteria will be assessed for enrolment into all domains that are active at a participating site.

Frequent adaptive analyses will be performed to generate updated posterior distributions of the treatment effect for each intervention in each domain. These probability distributions will be evaluated against a range of prespecified statistical triggers for superiority, inferiority, noninferiority, equivalence, futility, and harm. The exact set of triggers, along with the applied thresholds or deltas for each domain, will be specified in each DSA. Where interactions between interventions in different domains are anticipated to be likely, the statistical models can allow evaluation of such interactions. If so, the DSA may set statistical triggers that incorporate the interaction or have the interaction listed as a prespecified analysis at the time of reporting.

Interventions within each domain may be evaluated within prospectively defined and mutually exclusive strata (subgroups) of participants, but where information regarding the treatment effect from one stratum may be used (via “borrowing”) to contribute to the analysis of the effect of that intervention in other strata. Interventions found to be inferior, harmful, or futile for a specific stratum are removed from use in that stratum, allowing new interventions, domains, or both to be introduced. If there are three or more treatment groups in a domain, a response adaptive randomisation algorithm may be used to preferentially randomise participants to interventions that appear to be performing better. Simulation studies will be conducted in conjunction with consideration of the introduction of new interventions, domains, or both into PROMPT to inform the specifics of the design of a domain (sample size, adaptive rules, randomisation algorithm, and statistical triggers to be implemented).

## Study governance

The study governance structure is designed to provide appropriate management of all aspects of the study, considering multiple stakeholders and factors, including representation from regions that are participating in the trial, availability of skills and expertise related to trial conduct and statistical analysis, and content knowledge regarding the interventions that are being evaluated.

**Fig 3:**
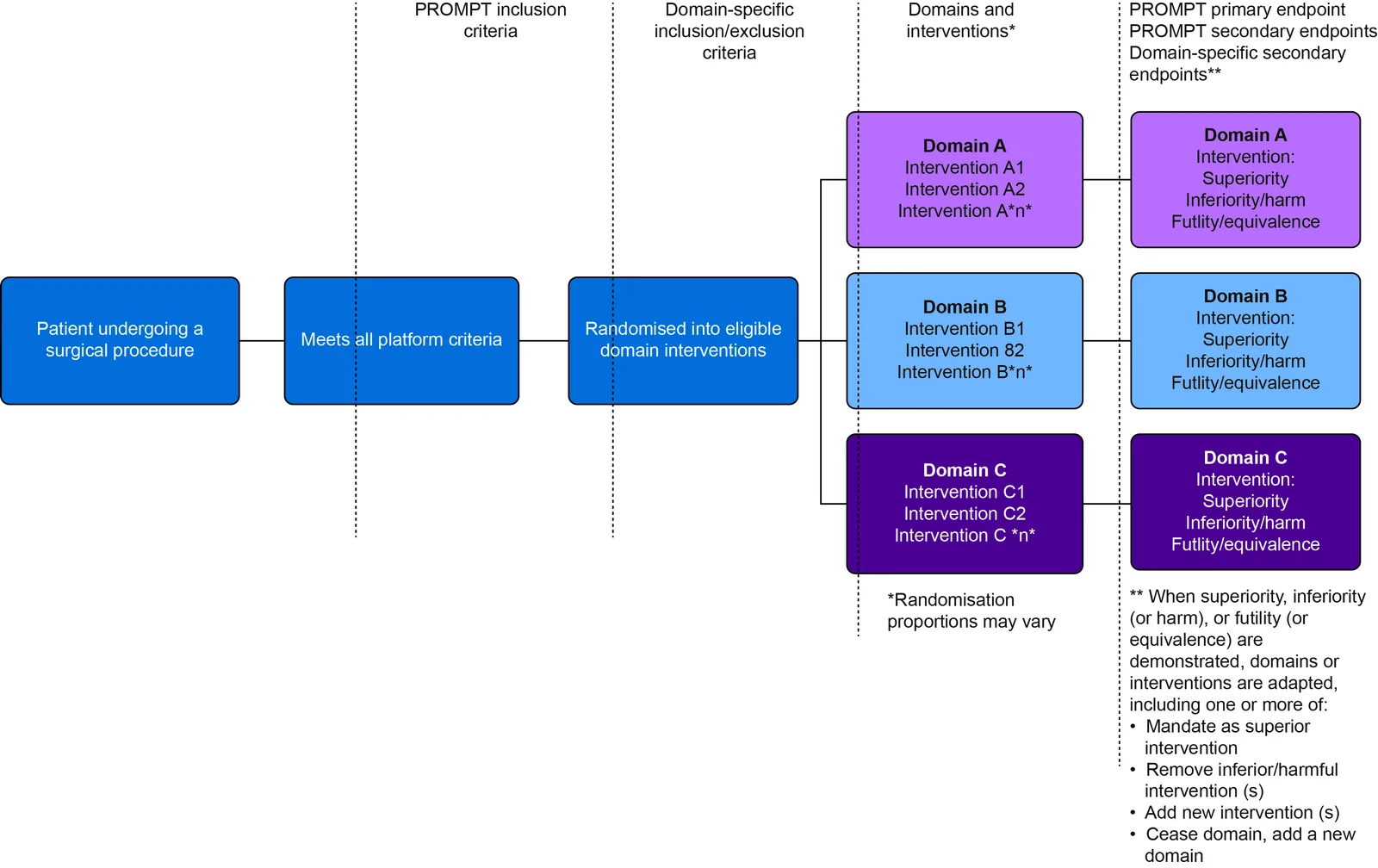
PROMPT governance structure. DSWG, Domain-Specific Working Group; PROMPT, perioperative medicine adaptive platform trial.

The Trial Steering Committee (TSC) is the key decision-making body and takes overall responsibility for the trial design, conduct, and reporting. Each participating region may have a Regional TSC that takes the primary responsibility for trial execution in that region. A Domain-Specific Working Group (DSWG) exists for each domain (or superdomain, comprising several domains that are closely related) and is responsible for the design and oversight of each domain. Each DSWG reports to the TSC. The Statistical Subcommittee and the Analytics team will have distinct responsibilities in providing blinded and unblinded information to the TSC and Data Safety Monitoring Committee (DSMC). The administration and governance structure for PROMPT is outlined in Fig. 3.

The TSC will seek and take advice from our Consumer-Stakeholder Advisory Committee, to facilitate patient and public involvement (consumer engagement) in the design, conduct, and reporting of the trial. Each DSWG should have at least two consumer representatives.

The responsibilities of the TSC are outlined in Table 2.

**Table 2:** The responsibilities of the Trial Steering Committee.

• Overseeing all governance and risk management.
• Executive decision-making based on recommendations from the data safety monitoring committee and other subcommittees and accounting for the interests of all trial participants and stakeholders.
• Development and amendment of the master protocol.
• Approval of all master protocol appendices (including domain-specific appendices, statistical appendix and other appendices, and their subsequent amendments).
• Recruitment and approval of new regions to PROMPT.
• Liaison with the data safety monitoring committee including, where appropriate, decisions regarding platform amendments and conclusions.
• Consideration of requests and approval of additional domains and their nested interventions to PROMPT including prioritisation of new domains, new interventions within a domain or both.
• Consideration of requests and approval of trial sub-studies.
• Liaison with the academic community including the International Committee of Medical Journal Editors regarding issues such as data sharing, governance and reporting of platform trials including PROMPT.
• In conjunction with data safety monitoring committee and domain-specific working groups, the communication and reporting of results from domains.
• Approval of manuscripts reporting results that are submitted by domain-specific working groups.
• Obtaining funding for PROMPT.
• Determining the strategic direction of PROMPT.

## Consumer-Stakeholder Advisory Committee

A Consumer group of people with lived experience of surgery will consider research priorities and planning, and advise on participant and public communications. Some people in this group will be members of the Consumer-Stakeholder Advisory Committee, which will also include representatives from medical professional bodies and policymakers. The Committee will work in accordance with its Charter (yet to be finalised), and in partnership with the research team, to inform key aspects of the study, including study design, selection of outcomes, participant materials, and dissemination of findings. Engagement will occur at defined time points throughout the trial lifecycle, and on an ad hoc basis, as required. Input from the Committee will be documented and considered by the study investigators to ensure that the trial is patient-centred, relevant, and aligned with community priorities.

## Role of the sponsors and funders

The sponsors and funders of PROMPT will have no role in the study design, data collection, data analysis, data interpretation, or writing of any reports.

## DSMC

Monitoring of the trial progress and recommendations concerning the overall conduct of the trial will be provided by a single DSMC for all participating sites and regions, which will regularly review data across all trial domains. The DSMC will operate under a Charter presented separately to this protocol, which will be approved by both the DSMC and the TSC before the commencement of the trial. The DSMC currently comprises five independent members with perioperative, infectious diseases, statistical, and platform trial expertise.

## Informed consent

In most cases, written informed consent will be obtained from all participants or their surrogate decision-makers before entry into the platform. Where an approved opt-out or alternative consent process applies, the site investigator is responsible for ensuring that it is conducted in accordance with the study protocol, ethics committee approval, and applicable regulatory and institutional requirements. There will be a single upfront consent process that covers PROMPT and all applicable domains. Typically, opt-in consent, when required, will be sought for each domain. Individuals may therefore consent to participate in one or more domains.

## Randomisation

PROMPT will randomly allocate participants to interventions for each domain that they are enrolled in. Where there are only two intervention options within a domain, that domain will be most efficiently powered by equal allocation to the interventions (1:1 ratio), thereby minimising the time needed to reach a trial conclusion for that domain.

## Trial conduct

PROMPT is designed to be embedded into usual clinical routines at participating sites, such that treatment decisions made by clinical staff can be replaced by randomisation in a streamlined fashion. Although not essential for participation in the trial, sites will be encouraged to develop processes that allow effective embedding of recruitment into normal workflow, with guidance provided by regional committees. Standard operating procedures will be developed at each site to guide clinical and research staff through the process of screening and randomisation.

## Study endpoints

Participants will be followed for 30 days after surgery for clinical endpoints/outcomes and economic outcomes (Table 3). Late notifications of SSI (e.g. prosthetic or deep space infection) up to 90 days after surgery will be included in the primary outcome because it is accepted that these begin within the 30-day follow-up period.

**Table 3:** PROMPT outcomes (full details are provided in the master protocol).

**Primary outcome**
• Incidence of superficial, deep and organ/space surgical site infection, including prosthetic (joint) infections, up to Day 30 (+7, if staff unavailable or participant not contactable in the preceding days) from index surgery, and including any late (up to Day 90) notifications of deep and organ/space SSI, or prosthetic (joint) infections.
**Secondary outcomes**
• Incidence of a composite of all other healthcare associated infections.
• Quality of recovery (QoR-15) at 3 and 30 days after surgery.
• Days alive and at home at 30 days (DAH_30_) after index surgery.
• A composite of major postoperative complications.
• All-cause mortality up to 30 days after index surgery.
• Quality of life (EQ-5D-5L) at 30 days after index surgery.
**Economic outcomes**
• Direct health care costs at 30 days after index surgery.
• Number of quality-adjusted life years (QALYs) at 30 days after index surgery.
• Cost effectiveness measured as a cost per QALY.
**Safety outcomes**
• Incidence of any prespecified safety outcomes as determined by the DSA, and adverse events.

## Bias control

Randomisation will occur for all domains; these randomisations are initially concealed, and an allocation is only deemed to have occurred if it is subsequently revealed to an administering member of the perioperative team. If surgery is deferred, attempts should be made to keep the participant in the assigned treatment group at that later time. Time zero (0) will be at induction of anaesthesia for surgery, when/if a preoperative or intraoperative intervention is being evaluated.

Allocation concealment will be maintained by using centralised randomisation, which is remote from study sites. The default position is that group assignment/treatment allocation will not be disclosed to the surgical team or trial participants.

The primary outcome of PROMPT (incidence of all SSI defined according to Centers for Disease Control and Prevention (CDC) definitions^18^) is not subject to ascertainment bias. Where possible, trial management personnel unaware of treatment allocations will conduct follow-up assessments. Where secondary endpoints are subjective, blinded outcome adjudication may be required and will be specified in the DSA as needed.

## Data Collection, management, and analysis

### Participant timeline

Participants will be followed for at least 30 days from index surgery to capture all relevant clinical and health economic outcomes.

### Source documents

Study sites must maintain study source documents. Source documents may include a participant’s medical records, hospital charts, the site investigator’s participant study files, and the results of diagnostic tests, such as x-rays and other imaging, and laboratory tests. The investigator’s copy of the case report form serves as part of the investigator’s record of a participant’s study-related data.

### Data collection methods

The processes for identifying outcomes will be conducted by the project research coordinator(s), who will collect patient characteristic data, relevant laboratory results, operative data, and quality of life (EQ-5D-5L). Active surveillance may comprise the following:

• Review of all participants’ medical records on discharge and follow-up.
• Web-based and/or telephone-based contact for outcomes and quality of life data at 30 days after index surgery.
• Contact with the participant’s general practitioner and other health care institutions, as required.
• In Australia, linkage to Medicare and pharmaceutical benefits data (and potentially equivalent data linkage in other countries, subject to availability).

### Web-based data entry

Date are entered into the database by research staff, after completion of the paper-based case report form or by direct entry of the data into the database through a secure web-based data entry system, monitored by the trial data management centre. All data fields are checked and automatically downloaded onto the database. All study personnel will have 24-h access to the study-coordinating centre to resolve any questions that may arise. Random audits of centres will be undertaken to assess the accuracy and legitimacy of the trial data. Statistical monitoring of the data completeness, data variability, and risk-appropriate endpoint rates will be done for all patient data.

### Schedule of assessments

Platform-wide patient assessments and data collection will occur before surgery, intraoperatively, and after surgery (Table 4). The extent and exact timing of these will be determined by each DSA. Primary, secondary, and safety outcomes will be detected by local research staff using direct patient follow-up visits, web-based or telephone-based patient contact, and medical record review.

**Table 4:** Data collection points in PROMPT.

**Baseline (preoperative)**
• Basic demographics, including sex, age, weight, medical history, history of smoking and medication history.
• American Society of Anesthesiologists physical status classification.
• Baseline investigations, as determined by the DSA.
• Baseline QoR-15 and quality of life measured using the EQ-5D-5L.
**Day of surgery**
• Operative data.
• Antimicrobial prophylaxis.
• Adverse events.
**Day 3, 5, 7 post-index surgery**
• Laboratory investigations (as part of standard care).
• QoR-15 (day 3).
**Day of discharge**
• SSI (primary outcome).
• Healthcare-associated infections.
• Other major complications.
• Adverse events.
**Day 30 (+7 days)**
• Any wound and other major complications since prior review.
• DAH_30_.
• QoR-15.
• EQ-5D-5L.

### Statistical analysis plan

The statistical models, approaches to reporting, adaptations, and decision criteria will be detailed in the statistical appendix to the master protocol and domain-specific statistical analysis plans (SAPs). Our current plan is provided in Supplementary Appendix S2.

Periodic interim (adaptive) analyses will be carried out using prespecified Bayesian models to monitor the accumulated trial data. Formal stopping rules will be evaluated at the adaptive analyses to determine whether to cease recruitment to answer a particular research question. The appropriate stopping actions arising from these rules will be triggered when the estimated statistical decision quantities (such as the posterior probability of observing a clinically important difference) exceed their predefined thresholds (boundaries). These stopping rules may result in the removal of an intervention arm from a domain, or the early stopping of a domain (or stopping recruitment to a stratum/subgroup within a domain). The adaptations that can be made will be domain-specific and conducted according to prespecified rules that will be used to reach conclusions regarding the effectiveness of the interventions under investigation. These adaptations may include early stopping of treatment arms for futility, inferiority, or harm; early stopping of a domain for superiority or futility; or changing the allocation ratios using response adaptive randomisation. Statistical triggers around noninferiority and equivalence may also be considered for some domains. The statistical decision quantities and statistical trigger thresholds will be defined using the investigators’ input and will ensure that suitable operating characteristics are produced (via computer simulations). Domain-specific values will be provided in the relevant DSAs/domain-specific SAPs. The results of the adaptive analyses will remain confidential unless a trigger has been met and a recommendation is made by the DSMC to the TSC that a conclusion has been reached and unblinding should occur.

Minimum sample sizes will be specified before performing any adaptive analyses for a particular domain (or for a particular comparison) and, in general, subsequent adaptive analyses will occur on a time basis (e.g. every 6 months). If a domain is slow to recruit (e.g. a small number of participants have been recruited since the last adaptive analysis), then the adaptive analysis for that domain may be delayed until sufficient number of participants have been recruited. If recruitment is much quicker than anticipated, then the frequency of the adaptive analyses may be revised.

Recruitment to a treatment group and/or a domain will be halted when an analysis reveals that a statistical trigger has been met, when a sample size cap is reached, or at the discretion of the TSC based on operational futility or emerging evidence from external trials. At this point, a final analysis of that intervention and/or domain will be carried out on the primary and secondary outcomes. Dissemination of intervention-specific results does not interrupt the platform trial; randomisation for new and other existing questions continues.

PROMPT will apply the estimand framework^19^ when analysing primary and secondary outcomes. This approach will specify the analysis population, the interventions being compared, the outcome of interest, the summary measure, and any intercurrent events (events occurring after randomisation that may preclude or influence interpretation of the outcome). Each domain will also have additional analysis sets for supportive analyses that consist of participants who were randomised to it and underwent induction of anaesthesia (or regional anaesthesia) for eligible surgery, and are analysed according to the treatment arm to which they were initially allocated, irrespective of any deviations from this treatment or any other protocol deviations. We may also perform per-protocol analyses (where appropriate), which includes participants who received or completed the treatment to which they were allocated according to the protocol. The primary endpoint and other binary endpoints will be analysed using Bayesian logistic regression. DAH_30_ will be analysed using Bayesian quantile regression, and death within 30 days of surgery will be coded as zero days at home. Continuous outcomes will be analysed using Bayesian linear regression.

The models will incorporate trial design features by accounting for potential heterogeneity by region, site, and time of enrolment (if needed). The models may also account for other covariates, such as whether the surgery was elective or nonelective, age group, ASA physical status, presence of diabetes or other strong prognostic variables, or any variables related to the randomisation scheme. The primary model will be a logistic regression on the primary outcome and incorporate model parameters that represent the effect of interventions assigned to participants within each domain. Secondary models may investigate interaction effects between interventions across different domains, and prespecified subgroup effect heterogeneity. The prespecified subgroups (for treatment effect heterogeneity) include age group, sex, ASA physical status, presence of diabetes, coronary artery disease, and elective/nonelective surgery. Exploratory analyses may be performed to augment the planned analyses (such as machine learning approaches to investigate treatment effect heterogeneity). Any *post hoc* or unplanned analyses will be clearly identified in any statistical reports and manuscripts for publication.

For adaptive and final analyses, the posterior distribution of the odds ratio associated with each treatment (relative to a reference treatment) will be estimated using Markov Chain Monte Carlo techniques, and the posterior mean, median, and 95% credible interval will be reported, along with the probability of the decision quantities of interest.

### Sample size calculation

PROMPT has an adaptive platform design, which means that it does not have a fixed sample size that determines when the study will end. The aim of PROMPT is to have perpetual recruitment until a conclusion can be made for all interventions under consideration and there are no new interventions to be investigated. Frequent analyses will be undertaken to assess the effectiveness of the interventions currently in the platform, and participants will continue to be randomised to those interventions, until there is sufficient evidence of superiority, efficacy, futility, harm, or inferiority of the intervention. Emerging evidence from external trials may also result in the removal of interventions if the TSC and the relevant DSWG judge the evidence to be sufficient to warrant closure.

### Health economic analysis

The conduct of a health economics analyses can occur after recruitment to a treatment group and/or a domain is halted. Whether a health economics analysis is conducted after halting of a single intervention in a domain will be determined by the DSWG in conjunction with the TSC, based on the rationale for a cost-effectiveness analysis in an ongoing domain. A detailed description of how the health economic analyses will be conducted for reporting the cost-effectiveness of interventions will be presented in the health economics appendix to the master protocol and the domain-specific health economic analysis plans, as appropriate.

### Adverse event reporting

All adverse events will be captured and reported as per the Australian National Health and Medical Research Committee (NHMRC) guidelines, and will be identified by system and severity. For this study, we will only be collecting adverse events that are considered to be related to intervention administration or procedure up to the day of discharge, after index surgery. Any serious adverse event (SAE) that is a component of the primary or secondary endpoints does not need to be reported separately as an SAE because it is being systematically captured and overseen by the DSMC and TSC.

### Ethics and dissemination

This study is to be performed in accordance with International Council for Harmonisation (ICH) GCP notes for Guidance on Good Clinical Practice, ICH E6(R3), and future updates. Chief investigators and associate investigators at study sites will oversee the implementation and conduct of the trial at their respective centres as the designated site investigator.

All PROMPT Research Staff will undergo initial training covering good research practice, research governance, and the relevant trial procedures. The trial procedures will be detailed in a manual. All these materials will be made available via the PROMPT website. Each site investigator must ensure that all staff conducting the study are qualified to do so.

## The PROMPT-O_2_ domain

PROMPT-O_2_ is the first domain in our platform trial. It is an international, multicentre, observer-blind, Bayesian adaptive randomised trial comparing three groups: liberal (FiO_2_ 0.8 [80%]) *vs* intermediate (FiO_2_ 0.5 [50%]) *vs* conservative (FiO_2_ ≤0.3 [30%]) oxygen delivery in patients undergoing major surgery. We will enrol up to 7800 patients throughout Australia, New Zealand, and our international collaborating sites (total >60 sites), all within our platform trial. The PROMPT-O_2_ domain-specific appendix is available at www.promptssi.org.

### Evidence Gaps – why we are doing this domain

The WHO recommendations state that “patients … should receive an 80% fraction of inspired oxygen (FiO_2_) intraoperatively” to reduce the risk of SSI (conditional recommendation; moderate quality of evidence).^2^ In contrast, lower concentration (more physiological) oxygen, which is titrated to achieve sufficient oxygen saturation during surgery, is endorsed by the World Federation of Societies of Anaesthesiology and the Thoracic Society of Australia and New Zealand. The US Centers of Disease Control and Prevention reviewed existing evidence and emphasised uncertain trade-offs between benefits and harms of increased FiO_2_, making no recommendations and declaring this an “unresolved issue.”^16^ The 2022 updated SSI prevention guidelines, sponsored by the Society for Healthcare Epidemiology of America, in collaboration with the Infectious Diseases Society of America, the American Hospital Association, and The Joint Commission considered the role of tissue oxygenation as “unresolved,” highlighting contradictory evidence from the most recent systematic reviews.^17^

## Dissemination plan

Data will be analysed and disseminated through peer-reviewed journals once a platform conclusion has been reached in a domain. The study will be reported using the Consolidated Standards of Reporting Trials guidelines and the extension for adaptive clinical trials.^20^ Findings will be made available through the PROMPT and other partner websites, as well as prepared for presentations at relevant scientific meetings.

## Endorsements

PROMPT has been endorsed by the Australian and New Zealand College of Anaesthetists Clinical Trials Network (ANZCA CTN) and the Australian Society of Infectious Diseases (ASID).

## Conclusion

PROMPT is designed to generate reliable information derived from real-world practice. The platform design allows the incorporation of new study arms that can evaluate any proposed improvements in care aimed at reducing SSIs and other serious complications after surgery.

## Authors’ contributions

Study conception and design: all authors

Wrote the first draft of the protocol: PM, TP, ER

Obtaining funds: PM, TP, DF

Development of protocol: PM, TP, ER, SW

Statistical expertise for the protocol: ER, AF, SW

Critical revision of the draft for important intellectual content and content relevant to participants and the community: all authors

Guarantor: PM

Read and approved of the final version of the manuscript to be published: all authors

## Study organisation and committees

*Sponsor:* Monash University, Melbourne, Australia.

*Funding sources:* Victorian Medical Research Acceleration Fund, Australian National Health and Medical Research Council, Health Research Council of New Zealand, and Monash University.

*Steering Committee:* Paul Myles (Co-Chair), Trisha Peel (Co-Chair), Elizabeth Ryan, Sophie K. Wallace, Wendy A. Brown, Tomas Corcoran, Tracey Bucknall, Tim Coulson, Daniel Frei, Brett Mitchell, Darshini Ayton, Alisa Higgins, Andrew Forbes, and Steve Webb.

*Statisticians:* Elizabeth Ryan and Andrew Forbes.

*Data and Safety Monitoring Committee:* Joshua Davis (Chair), Katherine Lee, Andrew Davidson, David Sidebotham, and Mark Smithers.

*Data Management and Quality Control:* Paul Myles, Sophie Wallace, and Adam Meehan.

*Consumer Advisory Committee:* Ricki Spencer (Chair), Sophie Wallace, Darshini Ayton, and Tracey Bucknall.

*Website Design and Maintenance:* Adam Meehan.

## Funding

Victorian Medical Research Acceleration Fund, Australian National Health and Medical Research Council, Health Research Council of New Zealand, Monash University.

## Declaration of interests

The authors have no conflicts of interest to declare.

## References

[1] Umscheid C.A., Mitchell M.D., Doshi J.A., Agarwal R., Williams K., Brennan P.J. (2011). Estimating the proportion of healthcare-associated infections that are reasonably preventable and the related mortality and costs. Infect Control Hosp Epidemiol.

[2] World Health Organization (2018). https://www.who.int/publications/i/item/9789241550475.

[3] Magill S.S., Edwards J.R., Bamberg W. (2014). Multistate point-prevalence survey of health care-associated infections. N Engl J Med.

[4] Adaptive Platform Trials Coalition (2019). Adaptive platform trials: definition, design, conduct and reporting considerations. Nat Rev Drug Discov.

[5] Angus D.C., Derde L., Al-Beidh F. (2020). Effect of hydrocortisone on mortality and organ support in patients with severe COVID-19: the REMAP-CAP COVID-19 corticosteroid domain randomized clinical trial. JAMA.

[6] Myles P.S., Yeung J., Beattie W.S., Ryan E.G., Heritier S., McArthur C.J. (2023). Platform trials for anaesthesia and perioperative medicine: a narrative review. Br J Anaesth.

[7] McLennan S., Griessbach A., Love S. (2026). Barriers and facilitators of platform trials. JAMA Netw Open.

[8] Glasbey J., Webb S.A., Peel T., Pinkney T.D., Myles P.S. (2025). Global collaboration between platform trials in surgery and anaesthesia. Br J Anaesth.

[9] Myles P.S., Kurz A. (2017). Supplemental oxygen and surgical site infection: getting to the truth. Br J Anaesth.

[10] Young P.J., Hodgson C.L., Rasmussen B.S. (2022). Oxygen targets. Intensive Care Med.

[11] Frei D.R., Beasley R., Campbell D. (2019). Practice patterns and perceptions of Australian and New Zealand anaesthetists towards perioperative oxygen therapy. Anaesth Intensive Care.

[12] Frei D.R., Beasley R., Campbell D. (2023). A vanguard randomised feasibility trial comparing three regimens of peri-operative oxygen therapy on recovery after major surgery. Anaesthesia.

[13] Chu D.K., Kim L.H., Young P.J. (2018). Mortality and morbidity in acutely ill adults treated with liberal versus conservative oxygen therapy (IOTA): a systematic review and meta-analysis. Lancet.

[14] Frei D.R., Moore M.R., Bailey M. (2024). Associations between the intraoperative fraction of inspired intraoperative oxygen administration and days alive and out of hospital after surgery. BJA Open.

[15] Frei D.R., Young P.J., Myles P.S. (2024). Oxygen therapy during surgery: decisions need reliable data. Anesthesiology.

[16] Berríos-Torres S.I., Umscheid C.A., Bratzler D.W. (2017). Centers for Disease Control and Prevention guideline for the prevention of surgical site infection, 2017. JAMA Surg.

[17] Calderwood M.S., Anderson D.J., Bratzler D.W. (2023). Strategies to prevent surgical site infections in acute-care hospitals: 2022 Update. Infect Control Hosp Epidemiol.

[18] Centers for Disease Control and Prevention. Surgical site infection event (SSI) (2025). https://www.cdc.gov/nhsn/PDFs/pscManual/9pscSSIcurrent.Pdf.

[19] European Medicines Agency, ICH E9 (R1) addendum on estimands and sensitivity analysis in clinical trials to the guideline on statistical principles for clinical trials, 2020 https://www.tga.gov.au/resources/resources/international-scientific-guidelines-adopted-australia/ich-e9-r1-addendum-estimands-and-sensitivity-analysis-clinical-trials-guideline-statistical-principles-clinical-trials (Accessed 17 August 2026).

[20] Dimairo M., Pallmann P., Wason J. (2020). The Adaptive designs CONSORT Extension (ACE) statement: a checklist with explanation and elaboration guideline for reporting randomised trials that use an adaptive design. BMJ.

