# Supplementary material for: A perioperative medicine platform trial (PROMPT) evaluating interventions to reduce the risk of infection and other major complications after surgery: rationale and design
Source: BJA Open. 2026 Aug 28;19:100855. doi: 10.1016/j.bjao.2026.100855 (PMC13545434; doi:10.1016/j.bjao.2026.100855)
Supplement: Supplementary file 1 [file mmc1.docx]

# **PROMPT Glossary**

This glossary contains definitions for terms that will be used throughout the PROMPT documents. The intention is for this glossary to be a centralised, living document that will be updated and improved upon throughout the duration of the platform trial.

The glossary incorporates terminology from the Australian Clinical Trial Alliance (ACTA) Innovative Trial Design Working Group “Innovative trial designs” glossary (which is currently being drafted), and from other platform trials (such as REMAP-CAP [[remapcap.org](https://www.remapcap.org/)]).

| **Term** | **Description** |
| --- | --- |
| Adaptive trial | A type of clinical trial design where aspects of the trial design may be modified according to pre-specified rules, typically based on analysis of accumulated data at interim analyses. Can be either Bayesian or frequentist. |
| Bayesian inference | A paradigm for conducting statistical analysis and inference that is governed by Bayes theorem and uses probability distributions to represent one’s current state of knowledge and uncertainty regarding the model parameter estimates (e.g., treatment effect) and to test hypotheses. It is often viewed as an alternative paradigm to “frequentist” (or classical) statistics.  From Gelman et al (2014):  “Bayesian inference is the process of fitting a probability model to a set of data and summarising the result by a probability distribution on the parameters of the model and on unobserved quantities such as predictions for new observations.”  Bayesian statistics is an approach to statistical inference that conditions on the observed data using a likelihood function and treats all other quantities, such as model parameters, as random variables. To perform Bayesian inference, the parameters of the associated likelihood function are assigned prior distributions. All inference is based on the *posterior distribution*, which combines information from the likelihood and prior distributions. That is, the posterior distribution summarises everything we currently know about the quantity of interest (e.g. treatment effect) |
| Bayesian hierarchical model | A type of statistical model that is implemented within the Bayesian framework that characterises a set of parameters from a common distribution where the parameters of the distribution are modelled in a hierarchical structure. The model allows parameter estimates (such as treatment effects) to vary across different (sub)groups but inferences on one parameter can depend on the empirical similarity to other parameters in the common distribution (e.g., the treatment effect estimate in one subgroup may depend on its similarity to the treatment effect estimates in other subgroups). |
| Borrowing | An informal shorthand for a Bayesian modelling technique that uses relevant information from other subgroups within a study/trial, typically via a hierarchical model, to provide a more informative estimate of efficacy in other trial subgroups of interest. (i.e., Inference may be improved by leveraging information across these different subgroups.) Borrowing may be *fixed* or *dynamic.*  *Fixed borrowing*: the amount of borrowing that occurs between subgroups is specified upfront.  *Dynamic borrowing*: the amount of borrowing that occurs between subgroups depends on the similarity of the subgroups. (Also known as *partial pooling*). That is, the study data inform the degree of borrowing based on the similarity of data between subgroups (and their sample sizes). If the treatment effects are very different little borrowing occurs; if the treatment effects are very similar, more borrowing occurs. |
| Concurrent controls | Trial participants that were recruited to the control arm over the same time period that the intervention/experimental arm under investigation was present in the study.    The concurrent controls for Intervention 3 are demonstrated in the figure above. |
| Contemporaneous cohort | Trial participants who were randomised within the same stage of an adaptive trial. |
| Cluster randomisation | When treatments are randomly allocated to groups (or clusters), rather than on an individual basis (as in *individually randomised trials*). Used as the basis for cluster randomised trials including stepped-wedge and cluster cross-over designs. In a cluster randomised trial, the ‘unit-of-analysis’ typically refers to the level at which the data are analysed. For example, the ‘unit-of-analysis’ may be at the individual level, but in some cases outcomes may be analysed at the cluster level. |
| Core Protocol | See *Master Protocol*. |
| Credible Interval | A quantity that is used to perform Bayesian inference: the interval within which the value of a population parameter of interest belongs with some probability. This can be used to represent the uncertainty around a parameter. An equal-tailed 95% credible interval (95%CrI) is typically used for inference, and says that “given the observed data, there is a 95% probability that the true parameter value falls within this interval.” |
| Current state | Also known as an implementation *guide.* This is an operational document that supplements the protocol documents and statistical analysis plans to provide additional details and the necessary trial status information required for a qualified statistician to be able to implement the pre-specified adaptive analyses. |
| Decision quantity | A measure of evidence that is used to make trial decisions, for example arm stopping in an adaptive trial. This quantity can be either Bayesian (e.g. uses the posterior probability or posterior predictive probability) or frequentist (e.g. a p-value). In PROMPT we will use Bayesian decision quantities.  Also known as a statistical quantity or quantity of interest.  E.g. posterior probability the OR < 1, i.e. Pr(OR<1 \|data), or posterior probability an intervention is the best in a domain, or posterior predictive probability the trial will be successful if it recruits to the maximum sample size. |
| Decision rule | A statistical rule that states the point at which a decision quantity passes a decision rule threshold, whereby a statistical trigger has been met and following the rule should be recommended to the Data Safety and Monitoring Committee (DSMC). Typically, decision rules are constructed to represent either superiority, equivalence, inferiority, or the non-inferiority of an intervention, or the statistical futility of continuing the trial to reach any of these conclusions. Can be either Bayesian or frequentist; PROMPT will use Bayesian decision rules.  e.g., drop an intervention arm for lack-of-benefit if there is a low (<5%) posterior probability of any benefit, denoted by Pr(OR<1 \|data) < 0.05 |
| Decision rule threshold | The specific threshold, applied to a decision quantity of interest, at which a decision rule is to be followed. For example, a nominal 99% threshold might be used to construct a Bayesian decision rule that requires a posterior probability of a treatment effect being positive to be greater than 0.99, or frequentist decision rule of a p-value less than 0.01.  Also known as the *statistical trigger threshold.* Referred to as “stopping boundaries” in some contexts (e.g. group sequential designs). |
| Domain | Consists of a set of competing alternative treatments that are of a particular treatment class (common clinical mode of action) or share a clinical context of use, which are mutually exclusive (see diagram below).    e.g. antibiotic domain, antiviral domain, analgesic domain, fluid management domain, exercise intervention domain.  A patient can only be randomised to one treatment arm within a domain, but can be enrolled in multiple domains and receive a treatment allocation for each domain that they are eligible for (see multifactorial platform trial). Domains may have additional eligibility criteria applied (in addition to the core platform eligibility criteria). |
| Domain-specific appendix (DSA) | An appendix to a core or master-protocol document describing the protocol relating to a given domain of a multifactorial platform trial. Each domain will have its own DSA. The information contained in each DSA includes criteria that determine eligibility of patients to that domain, the features of the interventions and how they are delivered, the relevant units-of-analysis, and any additional endpoints and data collection that are not covered in the Core Protocol. |
| Domain-specific working group | A sub-committee involved in trial management, the members of which take responsibility for the development and management of a current or proposed new domain. |
| Early stopping | The process of stopping a trial, or a component of a trial, on the basis of accumulated data and a decision rule, before the trial was originally planned to terminate. |
| Efficacy | A conclusion (statistical trigger) that an intervention leads to better outcomes compared to a reference (control) treatment. |
| Embedding | The process of integrating research activities within routine patient care, e.g., screening, recruitment, delivery of intervention, and data collection. |
| Enrichment | A study design that allows researchers to identify sub-populations of participants for whom a proposed intervention is more likely to be beneficial and increase enrolment from those sub-populations (and potentially halt accrual from sub-populations for whom the intervention is not showing benefit) |
| Epoch | An element of a set of mutually exclusive and exhaustive time periods that is typically used in the statistical modelling for clinical trials with response adaptive randomisation (or changing randomisation allocations) or long-running platform trials to either incorporate non-concurrent control groups or changing treatment dynamics (with respect to time). |
| Final analysis | Also known as terminal analysis.  A special type of scheduled analysis that occurs at the cessation of a trial, or a component of the platform trial (such as the closure of a domain or treatment arm). The results of such an analysis are typically made publicly available (whilst ensuring that the integrity of any remaining components of the trial is not compromised). |
| Futility | A statistical decision rule, or statistical trigger, at which point there is little to no chance of demonstrating a certain-sized treatment effect (e.g. a clinically-important effect, a large effect or any effect) if the trial were to continue as planned. |
| Group sequential design | A particular type of trial design where data are examined at interim analyses and decisions are made as to whether to stop or continue the intervention(s) under investigation, without inflating the type I error. |
| Harm | A conclusion (statistical trigger) that an intervention leads to a worse outcome than some reference (control) treatment. |
| Hierarchical model | A statistical model that allows for clustered data (e.g., repeated measures on individuals, or data from individuals that belong to a particular group). Also commonly described as “mixed effects”, “random effects”, “random intercept”, and “random slope” models and can be fit using frequentist or Bayesian approaches. |
| Inferiority | A conclusion (statistical trigger) that an intervention performs worse compared to other interventions/treatments. This comparison may or may not include a control. |
| Interim analysis | Also known as *adaptive analysis.*  A pre-planned analysis conducted over the course of an ongoing trial using the data accumulated so far. May be performed based on elapsed calendar time (e.g., every 6 months), or based on some recruitment/follow-up target (e.g. total number of participants recruited or with primary outcome data available). Results can be used to implement pre-specified decision rules and potentially modify the conduct of the trial. |
| Likelihood function | Describes the joint probability of the observed data as a function of the parameters of the chosen statistical model. |
| Live interventions/treatments | Treatments within a domain that have a non-zero randomisation probability (including standard-of-care/control). |
| Markov Chain Monte Carlo (MCMC) | A simulation technique used in Bayesian statistics to approximate the posterior distribution of parameters of interest and to sample from it. |
| Master Protocol | Also known as a *core protocol.* Is a module of the protocol documents that governs the trial and contains all information that is generic to the platform trial, irrespective of the regional location in which the trial is conducted and the domains or interventions that are being tested. The master protocol can be considered a “blueprint” for conducting the study.  The master protocol specifies:  - overall platform trial structure  - overall objectives/aims of the platform trial  - study population(s) and common inclusion and exclusion criteria across the interventions  - standardised trial procedures and core measures/outcomes collected  - primary analysis and decision rules (including rules for adaptations)  - trial governance structures  It is intended that the master protocol will remain relatively static over time, but the occasional updates may be required (and submitted for ethical approval). The master protocol is typically supplemented by appendices that describe more specific aspects of the trial and facilitate design adaptations. These additional appendices may include domain-specific appendices, regional appendices, and statistical appendices (adaptive design report/simulation appendices, statistical analysis plans, and an implementation guide/current state that describes how to perform the adaptive analyses). |
| Monte Carlo | A simulation technique that uses repeated random sampling and statistical models to evaluate the performance of a design (or a procedure) and study its performance. |
| Multifactorial platform trial | A multifactorial platform trial, also known as a multi-domain platform trial, is a type of platform trial design in which each patient is randomised to a single intervention from each domain to which they are eligible. i.e., patients can potentially receive multiple treatment allocations across different domains of treatment. (The interventions are mutually exclusive within a domain). Some well-known examples include REMAP-CAP, RECOVERY, PERIOP. |
| Multiple imputation | A statistical approach used to handle missing data. It aims to allow for the uncertainty around the missing data by creating several completed datasets where the missing values are imputed based on the observed data and appropriately combined parameter estimates are obtained. |
| Non-concurrent control | Data from trial participants assigned to a control arm of a platform trial/adaptive trial who were recruited before a specific experimental treatment has entered the platform trial. Non-concurrent controls may improve trial efficiency at the cost of increased bias due to time trends.    The nonconcurrent controls for Intervention 3 are demonstrated in the figure above. |
| Non-inferiority | A conclusion (statistical trigger) that an intervention does not lead to worse outcomes than a reference treatment by some pre-specified amount, called the non-inferiority margin. A one-sided version of equivalence. |
| Operating characteristics | The long-run properties of a clinical trial design, that are often obtained via simulation. These typically include the relationship between sample size and corresponding statistical power and likelihood of type I error. |
| Outcome adaptive randomisation | See *response adaptive randomisation* |
| Participant randomisation scheme | An individual listing that captures, for each participant, the treatments that were available to them and their chance of receiving those treatments. |
| Platform Conclusion | Describes a decision of the Trial Steering Committee (TSC) to conclude that superiority, efficacy, inferiority, equivalence, non-inferiority, harm or futility has been demonstrated following the occurrence of a statistical trigger that has been evaluated by the DSMC. Under most circumstances a Platform Conclusion leads to a Public Disclosure of the result via presentations and publication, and may result in interventions being removed from the platform or closure of a domain. |
| Platform trial | A trial design with multiple primary research questions conducted within a single trial/protocol and has the potential to add new questions to the ongoing trial by incorporating new treatments and/or subgroups.  Evaluates multiple treatments/interventions for a disease/condition within one or more domains, across one or more subgroups of participants, with the available treatments in the trial changing over time.  Often intended to be “perpetual”; permit new interventions to be added to the platform over time, even if they were not pre-specified at the initial design stage.  These studies are conducted within the same environment, utilising common infrastructure and resources. |
| Posterior distribution | The posterior distribution (or simply posterior) is used in Bayesian analysis to describe the information about a parameter of interest (e.g. intervention effect) after observing data. It is typically a combination of the prior distribution and new evidence provided by the likelihood function. It can be calculated at an interim analysis or any time an update is desirable. |
| Prior distribution | Also referred to commonly as a ‘prior’. Represents the investigator’s belief about the true value of a parameter, without knowledge of the observed sample data. May incorporate previous information (e.g. from previous studies). |
| Posterior predictive distribution | The distribution of unobserved (often future) data based on observed data and a Bayesian model (including a prior distribution). Often used in clinical trials to predict the results at the end of the trial and/or after completing follow-up of the recruited participants, based on the posterior distribution. |
| Regimen | Describes the combination of interventions, including controls, across one or more domains that a participant has been allocated in a multifactorial platform trial, with one intervention allocated per domain. |
| Response adaptive randomisation | A randomisation procedure that uses past intervention assignments and participants’ responses to alter the probability of allocation to different intervention arms/regimens. Typically favours better performing arms/regimens at the time the calculation is made. |
| Simulation | Computer-based experimentation to evaluate operating characteristics of trial designs. Involves generating large numbers of ‘hypothetical’ trials with each trial sampling from an assumed probability distribution on, for example, treatment effects, recruitment rates, and times-to-events. Trials designs such as response adaptive designs are typically too complicated to be designed using closed-form mathematical equations. Commonly simulated operating characteristics include type I error (the proportion of false positive successes under the null hypothesis) and power (the proportion of true positive successes under different treatment effects). |
| Stage | A time period in an adaptive trial in which the study design is fixed (e.g. between interim/adaptive analyses). |
| State | **State** and **Strata** (see *strata*) are patient or disease-specific characteristics that can be used to define disease/patient subtypes or subgroups. They can determine eligibility to domains and may have differential treatment effects.  **State** is a set of mutually exclusive and exhaustive categories, defined by participant characteristics (covariates) that are capable of changing during the time that a patient participates in the platform trial (e.g. illness stage, disease state (mild, moderate, severe)). A change of state may alter eligibility of the patient after they’ve enrolled in the trial and a new set of randomisations may be required. State can be used to define the unit-of-analysis. |
| Statistical Analysis Committee (SAC) | Consists of 1 or more unblinded statisticians that takes responsibility for the conduct of the pre-planned adaptations in the platform trial. This task generally consists of running predetermined statistical models at each adaptive analysis and providing this output to the DSMC. From a trial governance perspective, the Statistical Analysis Committee is under the supervision of the DSMC. |
| Statistical analysis plan (SAP) | A document containing detailed and prespecified instructions for the required analyses of trial data. Often a distinction is made between a terminal SAP (that is to be implemented for a final analysis at the conclusion of the trial or the conclusion of a component of a platform trial such as a domain) and an interim analysis SAP (to be implemented at an adaptive/interim analysis; often named the *implementation guide* or *current state*). In a platform trial, an SAP is generally specified for each domain: domain-specific SAP (DSSAP). See also *Statistical Appendix* and *current state.* |
| Statistical Appendix | Also known as the Master Statistical Analysis Appendix (MSAA). A comprehensive addendum to the core protocol that specifies, in general terms, the randomisation strategies and the statistical model(s) that will be used to analyse the trial data. Ideally this document will remain unchanged throughout the trial. This document differs from a SAP in that it only contains general modelling strategies that may be applicable across the platform, whereas a SAP will provide modelling strategies that are more specific to the data being analysed (e.g. domain-specific). |
| Statistical trigger | Occurs when a threshold has been crossed for a particular decision rule. Statistical triggers may involve decisions regarding efficacy, superiority, inferiority, equivalence, noninferiority, harm or futility of the interventions under investigation within the platform.   A Statistical Trigger applies to the smallest unit-of-analysis specified for a given domain, but may be reached in more than one unit-of-analysis for the same intervention at the same adaptive analysis. |
| Statistical trigger threshold | See *decision rule threshold*.  Thresholds are based on posterior probabilities (posterior probability levels) in the PROMPT platform. |
| Strata | **State** and **Strata** (see *state*) are patient or disease-specific characteristics that can be used to define disease/patient subtypes or subgroups. They can determine eligibility to domains and may have differential treatment effects.  **Strata** comprise a set of mutually exclusive and exhaustive categories (stratum) that are defined by patient characteristics (covariates) determined at or before the time of enrolment (baseline) and are unchanged throughout the trial (e.g. diabetic, immunocompromised, previous exposure to treatment x, age group). The relative effects of interventions may be differential across the stratum. |
| Subgroup | A mutually exclusive baseline population characteristic into which participants of a trial can be partitioned (e.g. adults and nonadults).  See *strata*. |
| Superiority | A conclusion (statistical trigger) that an intervention leads to better outcomes than the other treatments within a domain. This may or may not include the control in the comparison. |
| Traditional trial | Often used to refer to a standard two arm, parallel group, randomised controlled trial. |
| Therapeutic area/ super domain | An area of healthcare that aims to be optimised (e.g. antiseptics & antimicrobials, anaesthetic approaches, surgical approaches, nutrition/metabolic processes) and may consist of several domains (using the definition of domain mentioned above). |
| Unit-of-analysis | Within a platform trial the unit-of-analysis is the group of participants whose data are analysed together within the model for a particular domain. The unit-of-analysis can be all participants who have received an allocation status in that domain or a subgroup of participants who received an allocation status determined by their status with respect to one or more strata or states. Within a domain, the response adaptive randomisation can be applied to the unit-of-analysis. |
