# Supplementary material for: A perioperative medicine platform trial (PROMPT) evaluating interventions to reduce the risk of infection and other major complications after surgery: rationale and design
Source: BJA Open. 2026 Aug 28;19:100855. doi: 10.1016/j.bjao.2026.100855 (PMC13545434; doi:10.1016/j.bjao.2026.100855)
Supplement: Supplementary file 2 [file mmc2.docx]

 **PROMPTSSI.org.au**

**Master Statistical Analysis Appendix (MSAA)**

| MSAA Version | 1.0 |
| --- | --- |
| Date | 29 May 2026 |
| Master Protocol Version | 1.1 |
| ClinicalTrials.gov Identifier | NCT07186634 |
| Universal Trial Number | P-01 |
| Website | www.promptssi.org |
| Sponsor | Monash University |
| Lead Investigators  (co-chairs of Trial Steering Committee) | Professor Paul Myles Professor Trisha Peel |

**SAP Contributors**

Elizabeth Ryan, Andrew Forbes, Paul Myles, Trisha Peel, Sophie Wallace

# MSAA Version History

| **Version Number** | **Date** | **Changes** |
| --- | --- | --- |
| 1.0 | DD MMM 2026 | Original version |

# Approvals

| **Version Number** | **Date Approved** | **Approver** | **Signature of Approver** |
| --- | --- | --- | --- |
| 1.0 | 29 May 2026 | Prof Paul Myles |  |
| 1.0 | 29 May 2026 | Prof Trisha Peel |  |

Table of Contents

[MSAA Version History	2](#_Toc230881536)

[Approvals	2](#_Toc230881537)

[Glossary	6](#_Toc230881538)

[Abbreviations	6](#_Toc230881539)

[1.0 Introduction	7](#_Toc230881540)

[1.1	Background and Rationale	7](#_Toc230881541)

[1.2	Objectives	7](#_Toc230881542)

[1.3	Purpose of This Document	7](#_Toc230881543)

[1.4	Summary of Changes	9](#_Toc230881544)

[2.0	Trial Structure	9](#_Toc230881545)

[2.1 Trial Design	9](#_Toc230881546)

[2.2 Trial Population	10](#_Toc230881547)

[2.3 Treatment Domains	10](#_Toc230881548)

[2.4 Regimen	11](#_Toc230881549)

[2.5 Subgroups	12](#_Toc230881550)

[2.6 Randomisation	12](#_Toc230881551)

[2.6.1 Stratification	13](#_Toc230881552)

[2.6.2 Response adaptive randomisation (RAR)	14](#_Toc230881553)

[2.7 Sample size	14](#_Toc230881554)

[2.8 Framework	15](#_Toc230881555)

[2.9 Adaptive analyses and stopping guidance	15](#_Toc230881556)

[3.0 Endpoints, Analysis Populations and Estimands	16](#_Toc230881557)

[3.1 Primary outcome	16](#_Toc230881558)

[3.2 Analysis populations	16](#_Toc230881559)

[3.2.1 Primary analysis population	16](#_Toc230881560)

[3.2.2 Domain-specific analysis populations	17](#_Toc230881561)

[3.3. Estimand	17](#_Toc230881562)

[3.4 Secondary outcomes	19](#_Toc230881563)

[3.5 Covariates	19](#_Toc230881564)

[3.5.1 Region and sites	19](#_Toc230881565)

[3.5.2 Epochs (time of enrolment)	20](#_Toc230881566)

[4.0 Statistical modelling	20](#_Toc230881567)

[4.1 Primary Model	20](#_Toc230881568)

[4.2 Prior distributions	21](#_Toc230881569)

[4.2.1	Effects of interventions	21](#_Toc230881570)

[4.2.2	Effects of randomisation to a domain and domain ineligibility	22](#_Toc230881571)

[4.2.3	Effects of regions and sites	22](#_Toc230881572)

[4.2.4	Effects of covariates	23](#_Toc230881573)

[4.2.5	Effects of epoch	23](#_Toc230881574)

[4.3 Secondary Outcomes	23](#_Toc230881575)

[4.3.1 Binary Secondary Outcomes	23](#_Toc230881576)

[4.3.2 Continuous Secondary Outcomes	24](#_Toc230881577)

[4.3.3 Days alive and at home after index surgery	24](#_Toc230881578)

[4.3.4 Safety Analyses	24](#_Toc230881579)

[4.4 Sensitivity Analyses	24](#_Toc230881580)

[4.5 Missing data	25](#_Toc230881581)

[4.6 Model Estimation	25](#_Toc230881582)

[4.7 Software	25](#_Toc230881583)

[5.0 Statistical Quantities / Decision rules	26](#_Toc230881584)

[5.1 Probability of efficacy or superiority/benefit compared to another intervention	26](#_Toc230881585)

[5.2 Probability of harm or inferiority	26](#_Toc230881586)

[5.3 Probability of global superiority (optimal intervention within domain)	27](#_Toc230881587)

[5.4 Probability of global inferiority (worst intervention within domain)	27](#_Toc230881588)

[5.5 Probability of futility	27](#_Toc230881589)

[5.6 Probability of equivalence	27](#_Toc230881590)

[5.7 Probability of non-inferiority	28](#_Toc230881591)

[6 Trial adaptations	28](#_Toc230881592)

[6.1 Frequency and timing of Bayesian updates (interims/adaptive analyses)	28](#_Toc230881593)

[6.2 Trial adaptations	29](#_Toc230881594)

[6.3 Introducing new interventions	29](#_Toc230881595)

[6.4 Adaptations to allocation probabilities	29](#_Toc230881596)

[6.5 Decision processes (Platform Conclusions)	30](#_Toc230881597)

[6.6 Actions arising from platform conclusions	30](#_Toc230881598)

[7.0 Reporting	32](#_Toc230881599)

[7.1 Blinding	32](#_Toc230881600)

[7.2 Model Parameter Summaries	32](#_Toc230881601)

[7.3 Participant Progression	32](#_Toc230881602)

[7.4 Participant characteristics	33](#_Toc230881603)

[7.5 Protocol adherence	34](#_Toc230881604)

[7.6 Graphical and Descriptive Summaries	34](#_Toc230881605)

[7.7 Missing data	35](#_Toc230881606)

[The number and percentage of missing data will be reported for all baseline covariates and outcomes. When imputation is used, the variables with imputed values and the imputation method will be clearly documented.	35](#_Toc230881607)

[7.8 Changes to prespecified analyses	35](#_Toc230881608)

[References	36](#_Toc230881609)

# Glossary

See Master Protocol for the PROMPT Glossary.

# Abbreviations

| AE | Adverse event |
| --- | --- |
| ASA | American Society of Anesthesiologists |
| CDC | Centers for Disease Control and Prevention |
| CONSORT | Consolidated Standards of Reporting Trials |
| CRF | Case Report Form |
| CrI | Credible Interval |
| DAH | Days alive and at home |
| DAH_30_ | Days alive and at home to 30 days |
| DSMC | Data Safety and Monitoring Committee |
| DSA | Domain-specific appendix |
| DSSAP | Domain-specific Statistical Analysis Plan |
| EQ-5D-5L | EuroQol 5 Dimension 5 level |
| ESS | Effective sample size |
| FACTS | Fixed and Adaptive Clinical Trial Simulator |
| FAS | Full analysis set |
| HCAIs | Healthcare associated infections |
| HDU | High dependency Unit |
| ICU | Intensive Care Unit |
| MCMC | Markov chain Monte Carlo |
| mITT | Modified Intention-To-Treat |
| MSAA | Master Statistical Analysis Appendix |
| NDLM | Normal dynamic linear model |
| OR | Odds ratio |
| PP | Per protocol |
| PROMPT | Perioperative Medicine Platform Trial |
| QoR-15 | Quality of recovery -15 item questionnaire |
| RAR | Response adaptive randomisation |
| RSAs | Region-specific appendices |
| REMAP | Randomised, embedded, multifactorial (multi-domain) adaptive platform |
| SAE | Serious Adverse Event |
| SD | Standard deviation |
| SSI | Surgical site infection |
| TSC | Trial Steering Committee |
| VAS | Visual analogue scale |

# 1.0 Introduction

## 1.1 Background and Rationale

More than 313 million surgical procedures are performed worldwide each year, and surgical site infections (SSIs) remain among the most common serious postoperative complications, driving substantial patient harm and health‑system costs. Although many SSIs are preventable, uncertainty persists about the most effective prevention strategies in routine surgical care across diverse hospital settings and priority populations.

The Perioperative Medicine Platform Trial (PROMPT) is a pragmatic, embedded, multifactorial platform trial using a Bayesian adaptive design framework to rapidly generate actionable evidence to prevent SSIs. PROMPT will be conducted across multiple hospitals in several regions of the world. PROMPT will include participants undergoing a broad range of surgical procedures, including (but not limited to) abdominal surgery, cardiac surgery, orthopaedic surgery, obstetric and gynaecological surgery. Specific interventions under study will be described in domain-specific appendices (DSAs).

PROMPT uses a Bayesian analysis framework. A primary model (specified below) drives all trial adaptations and will be used in reporting the results for specific interventions and domains.

## 1.2 Objectives

The aim of PROMPT is to efficiently identify effective, ineffective, or harmful treatments to reduce the incidence of perioperative infections and other major postoperative complications

## 1.3 Purpose of This Document

PROMPT uses a modular protocol structure, typical of adaptive platform trials. Modules within the protocol include the Master Protocol, multiple domain-specific appendices (DSAs), future region-specific appendices (RSAs), a Health Economics appendix, and a Statistical Appendix. The Statistical Appendix consists of several appendices/documents: a Master Statistical Analysis Appendix (MSAA), Domain-Specific Statistical Analysis Plans (DSSAPs), Simulation Appendix/Appendices, and a “Current State”/ “Implementation Guide”. The structure of the PROMPT protocol is outlined in Figure 1 below.

This document (the MSAA) presents the general framework for the statistical design and analysis plan for PROMPT. It draws on information from the PROMPT Master Protocol, Domain-specific appendices (DSAs) to the Master protocol and relevant CRFs (current versions). This document defines the trial objectives, the core definition of the primary endpoint, generic statistical methods/models and population level estimators within the estimand framework, and generic statistical triggers and rules for adaptations.

The MSAA is designed to be generic and flexible to accommodate additional domains and interventions over time (as well as removal of interventions and domains) without the need for revisions to the document to accommodate these changes. To provide a stable description of the analytical approach over the duration of the trial (and minimise the need for revisions), the content of this document generally avoids referencing specific treatment interventions.

Domain-specific Statistical Analysis Plans (DSSAPs) will provide additional details on the specific modelling used, based on the general concepts outlined in this document, and the information in the relevant DSAs. The DSSAPs will also provide detailed descriptions of analyses that are specific to a domain (e.g., domain-specific endpoints, subgroups, the unit of analysis) and will specify any deviations from the analyses outlined in the MSAA, which they (the DSSAPs) will supersede where applicable. The DSSAPs also provide descriptions of the statistical triggers and decision rules used in the domain, along with the timings of the adaptive analyses. We also note that domain specific variations might be included in the “Statistical Considerations” sections of the DSAs.

The full and complete statistical model for the current trial state is described in a separate document, the Current State of the Statistical Model (“Current State Document”), that is modified to reflect the changing status of the trial. The Current State Document describes the current domains, interventions, strata, states and current statistical modelling at a particular point in time of the trial.

The Simulations Appendix (which also forms part of the Statistical Appendices) will be updated periodically as an operational document as new interventions and domains are added to the trial. The Simulations Appendix will contain the results of Monte Carlo simulations that are conducted to demonstrate and understand the operating characteristics of a domain or comparison of interest, across a range of plausible assumptions regarding the outcome rates, treatment effects, and recruitment rates.

Statisticians working on analyses for PROMPT must read this document in conjunction with the other statistical documentation to get a full understanding of the analytical approach. Whilst the MSAA is intended to undergo relatively little change over time, the Current State Document is specific to the current state and structure of the platform trial and is expected to change. Our statistical documentation hierarchy is designed to minimise duplication where possible and requires some cross‑referencing between documents. Higher‑level documents introduce core concepts, which can then be elaborated in lower‑level documents (such as the Current State Document), but as a general principle we avoid repeating content across documents where possible.

This appendix (MSAA) proceeds by describing, in general, the trial structure, randomisation strategy, primary outcome and estimands, statistical models, and the trial adaptations. Specific aspects of the design, including the interim/adaptive analysis schedule, maximum sample size (for a domain), decision rules, and domain operating characteristics will be summarised in a separate PROMPT Simulations Appendix as well as the DSAs and DSSAPs.

**Figure 1**. PROMPT protocol structure

## 1.4 Summary of Changes

Version 1.0 of this MSAA is based on Version 1.1 (4 December 2025) of the PROMPT Master Protocol.

At the time of finalisation of Version 1.0 of the MSAA, all contributors were blinded to treatment allocations and treatment-related study results at the time of their contributions.

This document was written and reviewed by those authors detailed in the Version History section.

# 2.0 Trial Structure

## 2.1 Trial Design

PROMPT is prospectively designed to be flexible; the flexible aspects of the design are planned and part of the Master Protocol. This study will use a randomised, embedded, multifactorial (multi-domain) adaptive platform (REMAP) trial design (Angus et al., 2020). It will investigate multiple interventions within multiple domains and multiple patient groups.

Each domain consists of a set of competing alternative treatments that are of a particular treatment class (common clinical mode of action) or share a clinical context of use, and are mutually exclusive. A patient can only be randomised to one treatment arm within a domain, but can be enrolled in multiple domains and receive a treatment allocation for each domain that they are eligible for. If interventions are given concurrently, interaction effects may be considered and will be pre-specified in the relevant DSAs and DSSAPs. The trial structure is depicted in Fig. 2.

**Figure 2.** PROMPT structure

PROMPT will begin with a single domain and will subsequently incorporate additional domains over time (subject to additional funding). It is intended that PROMPT will provide a perpetual infrastructure in which the number of domains and the composition of interventions within them may evolve over time, reflecting the removal of inferior/harmful treatments, the adoption of superior ones, and the introduction of new interventions of interest. The specific patient groups under investigation may also evolve throughout the trial.

## 2.2 Trial Population

The inclusion and exclusion criteria that define the target population for the platform are described in the Master Protocol; additional eligibility criteria apply for each domain and are described within the relevant DSAs.

## 2.3 Treatment Domains

A domain consists of a specific set of competing treatments that possess a common clinical mode, and treatments are mutually exclusive within a domain. Every domain-eligible patient will be randomised to one and only one of the available treatments in the domain. For generalisability, this document discusses the analyses in terms of generic domains without reference to the specifics, which are primarily detailed in the DSAs.

Using the notation in Maher et al. (2023), domains are denoted by *D_k_*, where *k* indexes a particular domain, and are contained with the set of all domains in the trial 𝒟:

{𝐷_𝑘_ ∈ 𝒟 ∶ 𝑘 = 1, 2, 3, ..., 𝑛_𝒟_},

where 𝑛_𝒟_ is the total number of domains. Interventions within a particular domain are labelled using a lower-case *d* that is indexed by a domain-specific subscript 𝑗 {𝑗 ∈ $J_{D_{k}}$ : $J_{D_{k}}$ = 1, 2, 3, ..., $n_{D_{k}}$}, where $n_{D_{k}}$ is the total number of interventions in domain *D_k_*. Under this nomenclature, *d_kj_* refers to intervention *j* within domain *D_k_*.

It is expected that the number of domains and the number of interventions within each domain will expand or contract as the trial progresses. If no arms are open to enrolment in a domain, then that domain will be considered closed.

Some domains will have a control arm which will be described in the relevant DSAs. Note that some domains may be comparative effectiveness studies that do not have a designated control treatment in the analysis and aim to compare all competing strategies/interventions to one another. The reference treatment for domain *D_k_* will be denoted *d_k1._*

Individuals who are not enrolled in domain *D_k_*, but are enrolled in other domains of PROMPT, will have a “treatment allocation” of *d_k0_* to denote “not randomised” or “no allocation” for that domain. This non-enrolment to a domain could be due to a number of site-level and/or participant-level factors, such as:

- The domain is not open for enrolment at the site where the participant is to be enrolled
- The domain is not open for enrolment (at any sites) at the time the participant is to be enrolled in the trial (participant enrolled prior to the domain start date; participant enrolled after the domain has closed)
- The participant is not eligible for the domain

A participant can only be randomised in PROMPT if, at their enrolling site, there is at least one domain (that they are eligible for) with two or more treatment options available to them. It is possible that only a subset of interventions is available within a domain (for domains investigating >2 interventions), and the participant can be randomised amongst the remaining interventions in the domain (provided there are at least two treatment arms available to them). The allocation probabilities must be re-weighted for this participant prior to randomisation (the details of which will be provided in the Current State Document).

Careful consideration is needed for the evolving nature of the platform trial, in which new domains or interventions may be introduced after recruitment has begun. Because previously enrolled participants were not randomised within these new domains, they cannot contribute to estimating relative effects within these domains; specifically, they will not serve as ‘control’ participants for the newly added domains but will receive the “not randomised” *d_k0_* assignment.

If only a subset of domains is available to a participant, then they can be randomised to treatments within the available domains. As these participants are excluded from contributing to the relative comparisons within the other domains (that they are not enrolled in), their individual likelihood will include a “not randomised” parameter for each excluded domain. Clearly, while a “not randomised” arm *d_k0_* is accommodated within one or more of the other domains, participants should not be thought of as having been randomised to these arms.

## 2.4 Regimen

We use the word *regimen* to describe the complete set of treatments that an individual is randomised to, across each domain in which they were randomised.

## 2.5 Subgroups

Patients in the platform may be categorised into groups based on some mutually exclusive characteristics, and for which subgroup-specific estimates of treatment effect are of interest (e.g. those living with diabetes vs those without diabetes; surgery type). Category memberships can be either fixed at platform enrolment (“Strata”) or dynamic over the course of participation in the platform (“State”; e.g. illness stage, disease severity (mild, moderate, severe)). Domain efficacy can be assessed separately for these subgroups, either by stratification or modelling, and may be applied by domains as inclusion or exclusion criteria, and to define analysis populations. Details will be stated in the relevant DSAs.

When separate treatment effects for subgroups are of primary interest, the membership categories of these subgroups may be used to form analytic strata, and the treatment effect of an intervention is modelled as varying across the subgroups. Analytic strata are specific to each domain. For example, presence of diabetes may be an analytic stratum for one domain but not for another. Treatment effects within each analytic stratum may be estimated independently or with dynamic borrowing to share information across stratum. The number of analytic strata may be expanded, or the existing strata may be modified as the trial progresses. The full specification of analytic strata and modelling will be detailed in the Current State Document based on all DSAs.

When treatment effect heterogeneity is of secondary interest, subgroup analyses will be performed that investigate interaction effects between the interventions in a domain and the pre-specified subgroups. The default pre-specified subgroups include age group (18-50, 51-65, 66-75, 76-85, 86+), sex, ASA physical status (1/2, 3, 4), presence of diabetes, presence of coronary artery disease, and elective/nonelective surgery. Note that for certain domains, estimation of separate treatment effects for some of these subgroups may be of primary interest and these subgroups may instead be used to form analytic strata (as mentioned above). Other domain-specific subgroup analyses may be specified in the DSAs. Subgroup analyses (whether of primary or secondary interest) must be pre-specified in a DSA and/or DSSAP.

## 2.6 Randomisation

In this trial, randomisation will occur for all domains. A single upfront consent process is used that covers PROMPT and all domains. Opt-in or simplified consent will be sought for each domain; individuals may therefore consent to participate in one or more domains. Randomisation will occur at the patient level once all information required to confirm eligibility for the platform and at least one domain has been entered into the secure randomisation website. After eligibility is confirmed, participants will be allocated to a treatment group through a password‑protected secure website using a central randomisation system that is remote from study sites, and treatment assignments will generally remain concealed from both the surgical team and trial participants. Some interventions must be disclosed (unblinded) to one or more members of the perioperative team.

At platform entry, participants will be randomised for all potential domains that they are eligible and have provided consent for, according to allocation probabilities detailed in each DSA and stratified by strata where required. Randomisations are initially concealed, and an allocation is only deemed to have occurred if it is subsequently revealed to an administering member of the perioperative team. A participant is only deemed to have entered a domain once an allocation has been revealed within that domain. Sites will receive a participant’s allocation to an intervention within a domain once a participant is confirmed eligible for that domain and, ideally, close to the time when the intervention is planned (most often on the day of surgery. Time zero (0) will be determined within each DSA but typically will be at the commencement of anaesthesia for surgery when a preoperative or intraoperative intervention is being evaluated. (Note that future domains may potentially investigate post-operative interventions, and alternative time zeros may be required for those domains.)

A participant may not be enrolled in a specific domain because of contraindications (e.g. allergies, intolerances, severe adverse events) or other reasons (e.g. lack of access, clinician discretion, site opted out of domain, domain not yet activated at time of enrolment, domain closed at time of enrolment). A participant may also be ineligible for certain interventions within a domain, or a site may opt out of participating in certain interventions within a domain (the site must still participate in two or more treatment arms to participate in a domain). Randomisation can be implemented for participants who are eligible to receive two or more treatment arms within a domain. Where a participant is eligible for only one option within a domain, the provision of a treatment “allocation” status is made (using the *d_k0_* assignment). The PROMPT database will record the eligibility of participants for each domain (and intervention-specific ineligibilities where appropriate) and will also record whether a participant was randomised to a treatment in each domain.

Domain randomisation status will be accounted for by including covariate terms in the statistical model that represent the incremental effect on the outcome of being randomised vs not randomised. Eligibility status and availability of a domain at the site and time of enrolment of the participant may also be accounted for in the statistical models by including covariates that encode these factors (e.g. 1. Domain not active or not available at this site (and thus participant not assessed for eligibility), 2. Participant not eligible, 3. Participant eligible). If a participant is ineligible for all interventions within a domain and/or an entire domain is unavailable at the participant’s site at the time of randomisation, then the participant will also be deemed as not being randomised in that domain.

Randomisation is performed separately for each domain, and the resulting combination of interventions is the patient’s regimen. In general, randomisation in a specific domain will be independent of other domains’ treatment assignment(s), except in cases where the randomisation is dependent on other domains, which will be specified in the relevant DSAs.

Randomisation will be performed using the mass weighted urn (Zhao et al., 2015) to ensure accurate allocation targets; this algorithm can be used for equal randomisation or with response adaptive randomisation (RAR). The specific randomisation procedures for each domain (e.g., fixed allocation ratios or RAR are detailed in the DSAs.

### 2.6.1 Stratification

Unless stated otherwise in the DSA, randomisation will be stratified by site. Any additional strata will be outlined in the relevant DSA. Randomisation is performed separately by analytic strata in that the randomisation probabilities to the interventions may vary depending on the group membership of the patient within the analytic strata.

### 2.6.2 Response adaptive randomisation (RAR)

Where there are only two intervention options (i.e., two treatment arms) within a domain, that domain will be most efficiently powered by equal allocation (1:1 ratio) to the interventions, and so equal randomisation will be used (with stratification) for domains with two treatment arms.

For domains with three or more treatments, we may consider using RAR, based on ethical, clinical, and statistical considerations. For statistical considerations, simulations will explore whether RAR will result in meaningfully greater efficiency and/or an expected reduction in patient harm.

For domains utilising RAR, as a default, equal randomisation will be used prior to any adaptive analyses. This initial “burn-in” period will be set for each domain. Following the first interim analysis, the allocation probabilities will be updated on a regular basis. The RAR algorithm and settings used will be detailed in the relevant DSAs/DSSAPs. The RAR (if implemented in the domain) will occur centrally as part of the computerised randomisation process. Sites will receive the allocation status and will not be informed of the randomisation proportions.

Where RAR is implemented, patient- or site-specific issues may result in one or more interventions within a domain being unavailable or inappropriate for a particular participant. In this case, provided domain eligibility is retained through the availability of at least two treatments, for a given participant the randomisation proportions will be adjusted for the intervention that a participant is eligible for.

## 2.7 Sample size

PROMPT has an adaptive platform design which means that it does not have a fixed sample size that determines when the study will end. The aim of PROMPT is to have perpetual recruitment until a conclusion can be made for all interventions under consideration and there are no new interventions to be investigated, or the condition itself is no longer a public health concern. Frequent analyses will be undertaken to assess the effectiveness of the interventions currently in the platform, and participants will continue to be randomised to those interventions until a statistical trigger is met that results in a platform conclusion. Emerging evidence from external trials may also result in the removal of interventions if the Trial Steering Committee (TSC) and the relevant domain-specific working group judge the evidence to be sufficient to warrant closure.

Individual domains may have fixed caps on sample size as described within their DSAs. The required sample size and operating characteristics for each domain will be determined within a Bayesian statistical framework via extensive Monte-Carlo simulations, involving sensitivity analyses that consider a range of plausible scenarios regarding effect sizes, recruitment rates, and baseline SSI rates. Simulations may be updated when a new intervention is added within a domain or whenever a new domain is added to PROMPT. The results of these are provided in the Simulations Appendix.

## 2.8 Framework

Inferences in this trial will be based on a Bayesian statistical model, which estimates the posterior distribution of the treatment effects of interest, based on the accumulating data in the trial and pre-specified prior distributions. Study conclusions may be made separately for each subgroup (state or strata, as appropriate) within each domain where applicable (as specified in the relevant DSAs). Where appropriate, we will use Bayesian hierarchical modelling to dynamically integrate information across subgroups (states or strata) to improve our ability to estimate the subgroup-specific and domain-specific treatment effects. This borrowing can result in an increase in statistical power, while allowing for subgroup-specific treatment effects, and attenuate extreme results in smaller subgroups.

## 2.9 Adaptive analyses and stopping guidance

PROMPT will operate within a Bayesian adaptive design framework, using pre‑specified statistical triggers to reach conclusions about superiority, efficacy, equivalence, non‑inferiority, inferiority, harm, or futility, as detailed in the DSAs and DSSAPs. Interim adaptive analyses will be conducted at scheduled intervals using Bayesian models and decision rules to evaluate accumulating data. Minimum sample sizes will be set before any adaptive analysis can occur for a domain (specified in DSA/DSSAP), and the timing of subsequent analyses may be adjusted depending on recruitment rates (as a default, they will occur 6-monthly).

At each adaptive analysis, formal stopping rules will be applied to determine whether recruitment to a treatment arm, subgroup (if applicable), or entire domain should cease. These rules are triggered when key statistical quantities, such as posterior probabilities of clinically meaningful differences, cross predefined thresholds. Possible adaptations include dropping arms for futility, inferiority, or harm; stopping a domain early for superiority or futility; or modifying allocation ratios via RAR. Non‑inferiority and equivalence triggers may also be used where appropriate.

Recruitment to a treatment arm or domain will cease when a statistical trigger is reached, a sample size cap is reached, safety issues emerge, or the TSC judges that operational futility or external evidence justifies stopping. Once a platform conclusion is reached for a domain, follow‑up for already‑randomised participants will continue, and final analyses will be completed, while the broader platform continues to randomise participants to other active questions without interruption.

Generic decision quantities/adaptation rules are outlined in Section 5 below, with domain‑specific parameters defined in the relevant DSAs and DSSAPs.

# 3.0 Endpoints, Analysis Populations and Estimands

## 3.1 Primary outcome

The primary outcome is the incidence of an SSI (superficial, deep, and organ space) (yes, no), as defined by the CDC (Berrios-Torres et al., 2017), up to 30 days after index surgery or while alive (if death occurs before 30 days). That is, those that die prior to 30 days without an SSI will be included in the analysis population (as “no SSI”). Late notifications of SSI (e.g. prosthetic or deep space infection) up to 90 days after surgery will be included in the primary outcome. It is assumed that these late SSIs (between 30 and 90 days post-surgery) occurred within the first 30 days but only became clinically detected beyond 30-days.

This is the primary outcome for trial results for each domain, unless otherwise specified in the DSA. While the primary outcome is the same for all patients and domains, it should be noted that objectives, populations, summary measures, treatments, intercurrent events and supplementary analyses will almost certainly differ from one domain to another and will of necessity be described in detail in the DSA and DSSAP documentation.

## 3.2 Analysis populations

The analysis set for a domain’s analysis will be specified in the DSSAP documentation using the populations defined below in conjunction with the estimand framework (European Medicines Agency, 2020). A summary of the dataset definitions is provided in Table 1.

### 3.2.1 Primary analysis population

The primary analysis population / full analysis set (FAS) consists of all participants randomised to an intervention in at least one domain in PROMPT who undergo induction of anaesthesia (or regional anaesthesia) for eligible surgery and are analysed according to the regimen to which they were initially allocated irrespective of any deviations from this regimen or any other protocol deviations. In general, we will use the recommendations outlined in Fergusson et al. (2002) to determine post-randomisation exclusions. Note that participants are only deemed to have a randomised allocation in a domain if their allocation was “revealed” (see Section 2.6). To be included in this population, participants must have reached the 30 days post-surgery time point with their primary endpoint status either known or known to be missing. Participants that have reached follow up, but for whom information has not yet been collected/entered into the database will be treated as missing until the data has been entered. Participants who have been randomised, but have not yet reached the primary endpoint, will be excluded from this analysis population. The primary analysis population will inform the primary estimand (see Section 3.3).

The final analysis (for a platform conclusion) will only use data for those participants who were enrolled into the platform prior to the platform conclusion. Where withdrawal occurs, the reasons will be documented in the participant CRF. All data collected until the time of withdrawal will remain part of the study data set and included in analyses. Further detail on the analysis sets can be found in the Current State Document.

### 3.2.2 Domain-specific analysis populations

In general, each domain will have additional analysis sets for supportive analyses that consist of participants that were randomised to a treatment within that domain and underwent induction of anaesthesia (or regional anaesthesia) for eligible surgery and are analysed according to the treatment arm to which they were initially allocated irrespective of any deviations from this treatment or any other protocol deviations

Other analysis populations may be used for secondary outcomes, in supportive analyses of efficacy endpoints (once a platform conclusion has occurred) or in the analyses of domain-specific safety endpoints (where applicable). Each DSSAP will detail the analysis population that will be used for each endpoint, and the type of analysis it forms (primary, secondary, sensitivity, subgroup).

Per protocol analyses may also be performed (where appropriate) which include participants who completed the treatment to which they were allocated as defined in the protocol (i.e., will only include those participants with no protocol deviations prior to the primary endpoint). As the interventions are defined within each of the DSAs, the per protocol population will be defined in the relevant DSSAPs. Some domains may not include a per protocol population or analysis due to the complexity of the domain protocol and/or the interventions. DSSAPs will also detail any relevant safety populations.

Intervention availability may change over time, and individuals may be ineligible to certain interventions within a domain. Several analysis populations may be of interest to certain domains for sensitivity analyses (e.g. those eligible for both a certain intervention and control in a multi-arm domain); these will be specified in the relevant DSAs/DSSAPs.

**Table 1.** Dataset definitions for use in analyses.

| **Analysis population** | **Description** |
| --- | --- |
| Primary analysis population | Full analysis set, consisting of participants randomised to an intervention in at least one domain in PROMPT who undergo induction of anaesthesia (or regional anaesthesia) for eligible surgery |
| Domain-specific analysis population | Subset of the full analysis set, consisting of the PROMPT participants enrolled in the current domain undergoing analysis and randomised to a treatment within the domain |
| Domain-specific per protocol population | Subset of the domain-specific analysis population that did not have a protocol deviation |

## 3.3. Estimand

PROMPT will incorporate the estimand framework (European Medicines Agency, 2020) in the analysis of primary and secondary outcomes. An estimand is a precise description of the treatment effect reflecting the clinical question posed by a given clinical trial objective. It has five attributes: population, treatment, outcome/endpoint of interest, summary measure, and possible intercurrent events (defined as an event that can occur post-randomisation and preclude or affect the interpretation of the outcome). Note that unless otherwise specified, all platform endpoints are measured from Day 0 (day of surgery).

The following intercurrent events are currently being considered for the platform-level outcomes (note that any deviations in specific domains will be explicitly outlined in the DSAs/DSSAPs):

- Cancellation or postponement of surgery
- Death
- Non-adherence to allocated treatment (which includes incomplete/underdosing, treatment crossover/switching, non-adherence to the assigned intervention and treatment discontinuation).

The estimand related to the primary outcome is displayed in Table 2.

**Table 2.** Estimand for the PROMPT primary outcome

| **Objective** | **Type of endpoint** | **Interventions** | **Population** | **Endpoint** | **Intercurrent event(s) and the proposed strategies to handle the intercurrent event(s)** | **Population-level summary measure** |
| --- | --- | --- | --- | --- | --- | --- |
| To estimate the effect of interventions in patients undergoing surgery (requiring a skin incision, expected duration of at least 2 hours and a planned overnight hospital stay) on the incidence of SSI (superficial, deep, and organ space), as defined by the CDC, up to 30 days after surgery | Primary: binary | Domain specific interventions (see DSAs) | Patients meeting the PROMPT eligibility criteria, randomised to a treatment in at least one domain | Incidence of superficial, deep and organ/space surgical site infection, including prosthetic (joint) infections, up to Day 30 (+7) from index surgery, and including any late (up to Day 90) notifications of deep and organ/space SSI, or prosthetic (joint) infections | 1. Cancellation or postponement of surgery – handled using the “principal stratification” strategy (since ineligibility would have occurred regardless of the treatment arm allocation) – “always surgery” patients* 2. Death – handled using the “while alive” strategy 3. Non-adherence to allocated treatment (which includes treatment crossover, non-adherence to the assigned intervention and treatment discontinuation) – handled using the treatment policy strategy | Odds ratio (OR) comparing each intervention to a reference (from an adjusted Bayesian logistic regression) |

*This is similar to how mITT is justified in surgery trials

The primary estimand will be the odds of the primary endpoint (SSI) for each treatment relative to a reference treatment at 30 days after index surgery for the primary analysis population. For most domains, the primary estimand is applied in the adaptive analyses.

Note that marginal probabilities of SSI for each treatment arm may also used for decision-making, along with absolute risk differences (e.g., may be used to form statistical triggers/decision rules for adaptive or final analyses). These will be obtained from the adjusted logistic regression models.

A secondary estimand that may be reported for some domains is the odds of the primary outcome for each treatment relative to a reference treatment at 30 days after index surgery for participants without protocol deviations. Primary endpoint data collected after protocol deviations have occurred will not be included in this secondary analysis.

## 3.4 Secondary outcomes

The core secondary outcomes are introduced in the Master Protocol and include dichotomous, continuous and skewed variables. Domain-specific secondary outcomes are given in the DSAs.

## 3.5 Covariates

We define covariates as baseline variables which are likely to have some prognostic value and/or are randomisation strata. Statistical models will account for key trial design features, including potential heterogeneity by region, site, and time of enrolment, and may also adjust for strong prognostic factors, as well as variables related to the randomisation scheme. For categorical covariates, in general, we will set the most frequently observed value as the reference value.

The following covariates are prespecified for inclusion in the primary analysis:

- Site
- Region
- Age group (18-50, 51-65, 66-75, 76-85, 86+)
- Elective or non-elective (expedited) surgery
- ASA physical status (1/2, 3, 4)

with the option to add further covariates as the platform evolves (such as time of enrolment). Additional covariates may be required for certain domains (e.g., surgery type (such as cardiac, orthopaedic, abdominal), comorbidities (such as diabetes)) and will be specified in the relevant DSSAPs. For example, if a new domain introduces entry criteria based on specific baseline characteristics, this categorisation may be incorporated into the model to adjust for differences between patients eligible and not eligible for that domain, ensuring fair treatment comparisons.

The current covariate modelling strategy will be detailed in the Current State of the Statistical Model Document. Additional sensitivity and supplementary analyses that may include additional covariates will be pre-specified in DSSAPs.

### 3.5.1 Region and sites

A *region* is defined broadly as either a geographical region or country. Sites are nested within regions/countries.

### 3.5.2 Epochs (time of enrolment)

The use of epochs (or time period adjustments) enables the trial to account for temporal changes in clinical practice or shifts in the underlying disease over the course of a multi-year study. In adaptive trials, failing to adjust for time of enrolment of the participant - particularly when treatment effects are estimated using control groups enrolled at different periods or when response adaptive randomisation is used - can introduce bias, inflate type I error, and/or reduce statistical power (Bofill Roig et al., 2022; Saville et al., 2022). It is therefore often important to explicitly incorporate time of enrolment into the analysis if the patient population changes over time (or if other secular effects occur). We expect this patient population to be slow-changing but make the provision to include these adjustments in the model if required. An epoch is defined here as the calendar time of enrolment of the participant corresponding to a pre-specified time period:

{*t* ∈ *T* : *T* = 1, 2, 3, ..., 𝑛*_T_*}

where the number *t* corresponds to the natural temporal ordering of epochs, and *n_T_* represents the most recent epoch period for the purposes of analysis. That is, there is one term for each epoch/time-period in the trial. The definition of these epochs will be provided in the Current State Document.

# 4.0 Statistical modelling

PROMPT data will be analysed using Bayesian statistical methods. The adaptations in the design are controlled by the statistical model. The modelling can expand and contract to accommodate the number of domains, interventions, strata, and states being evaluated at any time. Specific details of the model can be found in the Current State Document.

In general, the primary model described below will be used for primary analyses and at the adaptive analyses. However, some domains may require separate models, which will be detailed in the DSSAPs and Current State Document.

## 4.1 Primary Model

The primary analysis is a Bayesian logistic regression model for the binary primary outcome that is based on the primary analysis population (FAS; defined above). The model incorporates effects for all interventions across all domains within a single model and has the form:

$$\pi_{i}= {logit}^{-1}(\eta_{i})$$

$$\eta_{i} = \beta_{0} + \sum_{k\leq n_{D}} \sum_{j\leq n_{D_{k}}} \beta_{d_{kj}}I_{kj(i)}+\sum_{k\leq n_{D}} \gamma_{D_{k(i)}}R_{D_{k(i)}}+\sum_{k\leq n_{D}} \xi_{D_{k(i)}}NE_{D_{k(i)}}+\boldsymbol{z}_{i}^{T}\boldsymbol{\theta}+\boldsymbol{\delta}_{r(i)}+\boldsymbol{\omega}_{r(i),s(i)}+\boldsymbol{\varphi}_{t(i)}$$

where:

- $\pi_{i}$ is the probability of response (having an SSI) for participant 𝑖
- $\beta_{0}$ represents the baseline log-odds of response in the reference population
- $n_{D}$ is the number of domains
- $n_{D_{k}}$ is the total number of interventions in domain *D_k_*
- $\beta_{d_{kj}}$is the effect of the *j*-th intervention in domain *D_k_* compared to the reference treatment for domain *D_k_* (*d_k1_*)
- $I_{kj(i)}$ is an indicator for assignment to intervention $d_{kj}$ (intervention *j* within domain *D_k_*)
- $\gamma_{D_{k}}$ represents the effect of randomisation to domain *D_k_*
- $R_{D_{k(i)}}$ is an indicator for being randomised to domain *D_k_*
- $\xi_{D_{k}}$ represents the effect of ineligibility for domain *D_k_*
- $NE_{D_{k(i)}}$is an indicator for not being eligible for domain *D_k_*
- $\boldsymbol{\theta}$ represents the effect of other covariates specified for model inclusion
- $\boldsymbol{\delta}_{r}$ is the (fixed) effect of the region *r* in which the participant is located
- $\boldsymbol{\omega}_{r,s}$ is the effect of site *s* nested within region r, where site is included as a random effect using a hierarchical prior
- $\boldsymbol{\varphi}_{t}$ is the effect of the participant being recruited during epoch 𝑡

The parameter $\beta_{d_{kj}}$ is the log-odds of having an SSI for patients in the *j*-th intervention in domain *D_k_* compared to the reference treatment for domain *D_k._* This will be exponentiated to obtain an OR and is our treatment effect of interest. The model is parameterised such that an OR<1 (log-OR<0) represents benefit, and OR>1 (log-OR>0) represents harm. In general, each domain in PROMPT will contribute a set of additive treatment effects for the domain’s interventions. The exact form of the additive effects for each domain depends on the design features of the domain and are defined in the Current State Document.

For some domains, there may be redundancy between some of the eligibility variables and the strata variables/covariates that could create identifiability issues, and modifications of the model may be required in those instances.

Unless otherwise noted, it is assumed that there are no interactions between interventions across domains. Secondary models may investigate interaction effects between interventions across different domains; only two-way interactions will be considered. These will be pre-specified in the relevant DSAs.

## 4.2 Prior distributions

The following subsections outline the prior distribution structure for the parameters of the logistic regression model, along with some default values. The model for alternatively distributed endpoints will require a different prior structure, which will be described in detail in the DSSAPs that will be published prior to the closure of a domain for terminal analysis, and the Current State Document.

### 4.2.1 Effects of interventions

The prior for each intervention effect is defined in the DSSAP and Current State Document. In this section we describe default priors for the intervention effects that may be modified for each domain.

Within each domain, one intervention, typically the control or standard‑of‑care (if applicable) will serve as the reference, with its treatment effect fixed at zero. The effect of all other interventions in the domain will be estimated relative to the reference intervention.

The baseline response $\beta_{0}$ (intercept term) and treatment effects $\beta_{d_{kj}}$ (log-odds ratios) for domain-specific interventions *d_kj_* will be modelled using minimally informative normal prior distributions:

$$\beta_{0}\sim N(0,{\sigma_{0}}^{2})$$

$$\beta_{d_{kj}}\sim N(a,\sigma^{2})$$

where *a* is a fixed value set by the investigators. As a default we will consider ${\sigma_{0}}^{2}={2.5}^{2},$ *a* = 0 and $\sigma^{2}$ = 1.

### 4.2.2 Effects of randomisation to a domain and domain ineligibility

The log-odds ratios for the effect of being randomised to a domain ${(\gamma}_{D_{k}})$ and domain ineligibility ($\xi_{D_{k}})$ will be assigned independent normal prior distributions such that:

$$\gamma_{D_{k}}\sim N(a,\sigma^{2})$$

$$\xi_{D_{k}}\sim N(a,\sigma^{2})$$

where *a* and $\sigma^{2}$ are fixed values set by the blinded investigators. For the primary analysis, we will consider *a* = 0 and $\sigma^{2}$ *=* 1 as default values.

### 4.2.3 Effects of regions and sites

Region r=1 will be the reference region ($\delta_{1}=0)$ and the log-odds ratios for all other regions r = 2, …., R will have a standard normal prior:

$$\delta_{r}\sim N(0,1), r=2,...,R$$

The log-odds ratio of sites are nested within region hierarchically and will be treated as exchangeable within region such that:

$\omega_{r,s}\sim N\left( 0,\tau_{r}^{2} \right)$, s=1,…,S_r_, r = 1, …, R

A prior for $\tau_{r}^{2}$ will also be specified (described in the Current State Document). As a default we will use:

$\tau_{r}^{2}\sim Half-t(3,1)$

### 4.2.4 Effects of covariates

As a default, the log-odds ratios for other covariates will be assigned independent standard normal prior distributions such that covariate $z_{c}$ has the prior:

$$\theta_{Z_{c}}\sim N(0,1)$$

The parameter value for the reference category of a categorical covariate will be set to 0.

### 4.2.5 Effects of epoch

Temporal trends may be accounted for by splitting the trial sample into separate cohorts defined by contiguous epochs. In the early stages of the platform it is expected that there will be little change in the patient population, and time effects may be omitted in the first few runs of the primary analysis model. Over time this may be revised, and time effects may be modelled using categorical effects (with independent priors), or by using normal dynamic linear model (NDLM; first- or second-order) – approaches which have been suggested in the literature (Bofill Roig et al., 2022; Saville et al., 2022). The exact form of the model will be specified in the Current State Document (if these adjustments are required).

## 4.3 Secondary Outcomes

The primary analysis of each secondary outcomes will use the same model structure (linear predictor) as that used for the primary outcome. Binary variables will be analysed by logistic regression, continuous variables (with reasonably symmetric distributions) using linear regression, and DAH_30_ using quantile regression.

### 4.3.1 Binary Secondary Outcomes

The core binary secondary outcomes are:

- Incidence of a composite of all other healthcare associated infections (HCAIs) (pneumonia, blood stream infection and urinary tract infection)
- Incidence of a composite of major post-operative complications
- All-cause mortality up to 30-days after index surgery.

A separate Bayesian logistic regression model will be fit for each binary outcome. The outcome will be coded so that an odds-ratio < 1 implies treatment benefit. The model form will be:

$$logit(Pr(y_{i}=1))= \eta_{i}, i = 1,...,n$$

for participant 𝑖 with outcome $y_{i}$, and linear predictor $\eta_{i}$. The linear predictor, $\eta_{i}$, will take the same form as in the primary analysis model and the model will use the same priors. Sensitivity analyses may vary the form of the linear predictor.

### 4.3.2 Continuous Secondary Outcomes

The core continuous secondary outcomes are:

- Quality of recovery (QoR-15) score at 3 and 30 days after surgery
- Quality of life (EQ-5D-5L) at 30 days after index surgery

Continuous secondary outcomes (excluding DAH) with reasonably symmetric distributions will be analysed using multivariable linear regression; transformations may be used if required. The analysis will be adjusted for the baseline measurement of the outcome where available.

### 4.3.3 Days alive and at home after index surgery

Days alive and at home (DAH) will be calculated at Day 30 and/or at 90 [DAH30/90] post-index surgery. DAH will be calculated using mortality and hospitalisation data from the date of index surgery (=Day 0) until Day 30 or 90 (as specified in the DSA). DAH is calculated through subtracting the days from the index hospital length of stay, readmissions to hospital, or admissions to rehabilitation or other nursing facility (i.e. not at home). If the patient’s preoperative domiciliary status was in a nursing facility, then that can be counted as “home”. If the participant dies within the 30 or 90 days, the DAH score is zero (0).

DAH is typically a left-skewed distribution with a small spike at 0 days (bi-modal), due to early deaths. As a default, Bayesian quantile regression will be performed (alternatives may be required if there is a large proportion of deaths). DAH will be summarised with medians and interquartile ranges and with estimated differences between medians and their 95% CrIs computed; additional quantiles (e.g. 20^th^ or 25^th^ percentile) may also be reported (as specified in DSSAPs).

Other heavily skewed continuous endpoints will be analysed in a similar manner.

### 4.3.4 Safety Analyses

All reported AEs and SAEs will be summarised in aggregate and by intervention. Domain-specific safety outcomes will also be collected. The analysis of these outcomes will be specified in the DSSAPs along with other domain-specific outcomes.

## 4.4 Sensitivity Analyses

Sensitivity analyses for the core outcomes may be conducted. These analyses may involve varying the model structure and/or the analysis population to examine how robust the results are to different assumptions.

For instance, epoch and site parameters may be removed (set to 0), or the analysis may be limited to participants who were eligible for a specific intervention or domain. If an intervention was introduced after trial initiation, sensitivity analyses may also compare only those participants who were randomised concurrently. Additional sensitivity analyses may assess the impact of alternative prior specifications or different approaches to handling missing primary outcome data (e.g., worst‑case or best‑case scenarios).

The DSSAPs will specify the sensitivity analyses to be undertaken for each domain.

## 4.5 Missing data

A low incidence of missing outcome data (<1%) is expected. As a default we use the approach outlined below; any deviations to this will be noted in the Current State Document.

The primary analysis model will include all patients with at least 30 days of follow‑up; however, patients with no primary endpoint information (i.e., completely missing SSI data) will be excluded from the modelling. Missing primary outcome data will not be imputed, and participants without primary outcome data will be excluded from the adaptive analyses; for example, those who have reached the 30‑day endpoint but for whom outcome information has not yet been collected will be treated as missing. If a participant’s randomisation assignment is missing, they will be assumed to be ineligible for that domain, and if eligibility status is unknown, the participant will likewise be assumed to be ineligible for that domain or intervention.

Patients missing one item in the EQ-5D-5L or QoR-15 scales at any time point will have the value of the missing item imputed as the mean of the other items. Patients missing two or more items will have their EQ-5D-5L or QoR-15 scores set to missing. Patients who died before EQ-5D-5L or QoR-15 could be measured will have the corresponding endpoint set to missing.

Missing covariate information may be imputed based on other available data (e.g. missing region, site or time of enrolment). Otherwise, the modal value will be imputed for missing values.

## 4.6 Model Estimation

The Bayesian models will be updated using Markov chain Monte Carlo (MCMC) methods. For each parameter, we will run four or more MCMC chains in parallel for a ‘burn-in’ phase and sampling phase. Convergence and mixing of MCMC chains will be assessed using standard diagnostics, such as the effective sample size (ESS), R-hat, and graphical representations of the MCMC chains (e.g., trace plots). Parameters of the sampling algorithm may be adjusted to satisfy these diagnostics. The occurrence of divergent transitions will be attempted to be mitigated by increasing the target average acceptance probability rate and maximum tree depth to ensure that the sampler is exploring the parameter space fully.

## 4.7 Software

Analyses will be undertaken in specialised Bayesian software. Where possible, we will use MCMC methods implemented using the Stan software (Carpenter et al., 2016) to fit Bayesian models. Alternative software may be required for certain analyses (e.g., potentially for analysis of DAH_30_). Analyses will primarily be performed in R (version 4.4 or later; R Core Team, 2026). Additional analyses may be completed in Stata (v17 or later; StataCorp, 2021) if required.

Trial simulations to evaluate operating characteristics will be conducted either in R, using interfaces for Bayesian updating (such as rstan, rstanarm), or in specialised platforms such as FACTS (Fixed and Adaptive Clinical Trial Simulator; 2025).

# 5.0 Statistical Quantities / Decision rules

The trial’s decision criteria are based on posterior probability statements, which are evaluated at each adaptive analysis to guide trial adaptations, and may also be assessed at a final analysis following a platform conclusion. In PROMPT, these criteria rely on statistical quantities derived from the posterior distribution of model parameters, calculated using MCMC methods.

The decision quantities will be specified in each DSA/DSSAP and may be expressed in terms of odds ratios (OR) or risk differences (based on marginal estimates). A statistical trigger is deemed to have occurred if the condition(s) expressed in the decision rule has been met. For each decision rule specified below, we provide generic posterior probability thresholds $c$.

Each DSA/DSSAP will specify which statistical triggers apply to that domain, along with specific details of the decision quantities that will be used, and the actions that are to be taken once a statistical trigger has been met. Note that the thresholds used to define the decision rules will be determined via extensive computer simulations, the results of which are contained within the PROMPT Simulation Appendix.

## 5.1 Probability of efficacy or superiority/benefit compared to another intervention

For domains that include a placebo, standard-of-care or “no intervention” control arm, it may be of interest to compare the relative effectiveness of an intervention to that comparator to evaluate efficacy/effectiveness or harm.

We may also be interested in pairwise comparisons of interventions in a multi-arm domain, for example, in comparative effectiveness settings. In PROMPT, we may use the term “superiority” (“local superiority”) for reaching a conclusion (statistical trigger) that an intervention leads to better outcomes compared to another treatment in the domain.

The general form of the decision quantities used to declare efficacy or superiority for a given treatment arm is defined by assessing whether that intervention has a lower odds of the outcome compared with another arm within the same domain:

$$Pr(OR<1) > c_{sup}$$

5.2 Probability of harm or inferiority

Harm may be declared if there is a high probability that an intervention leads to a worse outcome than a control treatment (e.g., a placebo or “no active intervention” control). Similarly, inferiority of an intervention may be declared if there is a high probability that an intervention leads to a worse outcome compared to another (active) intervention. Decision quantities for harm/inferiority (compared to another treatment in the domain) may be of the form:

$$Pr(OR>1) > c_{harm}$$

## 5.3 Probability of global superiority (optimal intervention within domain)

In some settings, we may be interested in finding the best/optimal treatment within a domain (that may or may not contain a control). This may occur if the following decision rule is met:

$Pr(intervention d_{kj} is best in domain D_{k}) = Pr(d_{kj} = {argmin}_{J_{D_{k}}}({OR}_{J_{D_{k}}}) )> c_{opt}$

## 5.4 Probability of global inferiority (worst intervention within domain)

In some settings, we may be interested in removing the worst treatment within a domain (that may or may not contain a control). This may occur if the following decision rule is met:

$$Pr(intervention d_{kj} is best in domain D_{k}) = Pr(d_{kj} = {argmin}_{J_{D_{k}}}({OR}_{J_{D_{k}}}) )< c_{inf}$$

## 5.5 Probability of futility

In addition to looking at the probability of any level of benefit or lack-of-benefit, which calculate the probability that the difference between two treatments’ effects is below/above zero (OR<1, OR>1), the posterior probability that the difference between treatments’ effects falls into specific regions of interest may also be computed to evaluate futility, non-inferiority, or equivalence of two interventions.

Domains may specify futility rules that incorporate a clinically important treatment effect ${OR}_{fut}$ and declare futility of a treatment (compared to another treatment, often a control) if the following condition is met:

$$Pr(OR<{OR}_{fut}) < c_{fut}$$

Meeting this statistical trigger means that there is a low probability that the treatment will show a clinically important effect.

## 5.6 Probability of equivalence

Domains may specify an equivalence rule based on a predefined equivalence region for the odds ratio provided in the DSA. If two interventions within the domain have at least a $c_{equiv}$ posterior probability that the odds ratio comparing the two is with the prespecified equivalence region around 1, the two interventions will be considered equivalent:

$$Pr({OR}_{equiv}<OR<\frac{1}{{OR}_{equiv}}) >c_{equiv}$$

## 5.7 Probability of non-inferiority

Domains may specify non-inferiority rules using a predefined non-inferiority region for the odds ratio provided in the DSA:

$$Pr(OR<{OR}_{NI}) > c_{NI}$$

Note that if both equivalence and non-inferiority triggers are used in a domain, ${OR}_{NI}$ will generally be set to $\frac{1}{{OR}_{equiv}}$ (i.e. non-inferiority is a one-sided version of equivalence).

# 6 Trial adaptations

PROMPT is designed to be perpetual and answer both clinician and consumer priority-driven research questions. At scheduled intervals, the available data will be used to update posterior distributions that, in turn, will be used to inform operational decision rules based on prespecified statistical quantities (such as those specified in Section 5 above).

As the trial proceeds, some aspects of the trial status may change, for example randomisation probabilities may be updated, or treatment availability may change. Additionally, treatments and/or domains can be added or removed based on the trial results themselves, or due to information external to the trial.

## 6.1 Frequency and timing of Bayesian updates (interims/adaptive analyses)

Each domain/DSA will specify a minimum sample size that must be reached before any statistical triggers for that domain can be applied. Although data from domains that have not yet met this threshold may still contribute to the primary statistical model, for example, to adjust for other PROMPT domain treatments or to improve covariate estimation, no statistical triggers will be activated for those domains until the minimum sample size is achieved. Adaptive analyses are planned at 6‑month intervals following the initial minimum sample‑size analysis, though they may be skipped if enrolment is slow and little new information has accumulated, or brought forward if recruitment is rapid. These analyses will use all currently available data and will be conducted according to the current trial model as defined in the Current State Document. Interim primary analyses will include only participants who have completed 30 days of follow‑up.

Unblinded data will be extracted from the PROMPT database immediately prior to each analysis and provided to the PROMPT Analytic Team, who, once unblinded, will remain independent of all other trial committees. All patients enrolled in the perpetual trial contribute to the accumulating dataset, with a defined subset forming the primary analysis population. Participants remain in this population for the duration of the trial unless their data are deemed no longer informative due to expected temporal changes or because they are not contributing useful information to active domains.

## 6.2 Trial adaptations

The statistical quantities calculated based on the model run (model fit at an interim/adaptive analysis) will be used to trigger allocation updates and possible Statistical Triggers (determining superiority, harm, inferiority, equivalence, non-inferiority, etc.).

The following adaptations may be made at an adaptive analysis:

- Stop recruitment to a treatment arm within a domain
- Stop recruitment to a domain
- Update allocation probabilities

  These adaptations may be applied to all prospective participants to be randomised within a domain, or to certain subgroups, based on decision quantities that apply to all individuals within the domain or a subset of patients (based on analytic strata). The decision process for implementing statistical triggers is outlined in Section 6.5.

Other adaptations that may occur in PROMPT include:

- Adding a new intervention to an existing domain
- Adding a new domain to the platform

## 6.3 Introducing new interventions

While PROMPT is running, if a new intervention is introduced to an active domain (where an active domain is randomising amongst at least two treatments), then the randomisation will be “blocked” for the new intervention to guarantee an initial sample size. If there are $n_{D_{k}}$ interventions in a domain after the new intervention is started, then a fixed allocation of 1/$n_{D_{k}}$ may be used to allocate patients to the new intervention. The remaining 1−1/$n_{D_{k}}$ probability will be allocated to the other interventions using RAR or equal randomisation (depending on the randomisation scheme used for the domain). A certain duration of burn-in (using these allocation ratios) for each intervention will last until a certain number of participants have been allocated to the new intervention (detailed in DSAs/DSSAPs where applicable). At that point this restriction will be removed and RAR (or equal randomisation if applicable) to all treatments within the domain will be carried out.

## 6.4 Adaptations to allocation probabilities

If a statistical trigger indicates that a stopping rule has been met for a treatment arm, then that treatment arm will be removed from the domain (subject to a recommendation from the DSMC and approval by the TSC). Provided that there are two or more treatments remaining in a domain, then that domain will continue to recruit to the remaining treatment arms.

Certain domains with >2 treatment arms may opt to use RAR. After an initial burn in period, the allocation probabilities for the treatment arms within that domain will be updated at each adaptive analysis. The approach used to calculate the RAR probabilities will align with the objectives of the domain (e.g. find the best treatment arm quickest, find treatment arms that are superior to the control), and will be detailed in the relevant DSAs/DSSAPs. The RAR probabilities will be based on posterior probabilities, and may include measures to protect allocations to control (e.g. Trippa et al., 2012; Cellamare et al., 2017; Viele et al., 2020), to ensure that randomisation probabilities remain within a certain range (Wathen and Thall, 2017), or to weight the information based on the uncertainty of the measurement and/or the current sample size (Trippa et al., 2012; Cellamare et al., 2017).

Simulations will be used to inform the RAR algorithm/procedure that is to be used in a domain.

## 6.5 Decision processes (Platform Conclusions)

For adaptations internal to the trial, predefined rules are evaluated based on statistical decision quantities derived from the primary model and pre-specified thresholds (defined in Section 5). When a decision threshold is exceeded and criterion is met, the applicable oversight bodies (see Master Protocol) will consider the result and may determine that a platform conclusion has occurred for public reporting. For example, an intervention may be declared superior or harmful in a domain. In some situations, even when a statistical decision has been reached and a platform conclusion declared, the corresponding operational actions may be delayed. For example, if an intervention is deemed futile but additional data are still valuable for secondary outcomes, randomisation to that intervention may continue.

The following steps are involved in arriving at a platform conclusion:

1. An interim/adaptive analysis indicates that a criterion has been met

2. Results are reported to the DSMC for review

3. DSMC meet (as deemed necessary) and determine whether to report that a criterion has been met and recommend the implied action to the TSC.

If a platform conclusion has been declared and it has been decided that action is to be taken for that research question (e.g., closure of a domain) then the following additional steps may be performed:

4. All participants enrolled in the relevant analysis population(s) up until the time when the action was triggered are followed up

5. A final (platform conclusion) analysis is run on the completed follow-up for those enrolled participants (in the relevant analysis population(s))

6. The “final analysis” for that research question is conducted and reported.

For certain platform conclusions (such as harm or inferiority), a formal final analysis may not be performed for the treatment arm at the time of the statistical trigger, and final reporting may be delayed until closure of the domain, to prevent unblinding of results for ongoing treatments within the domain. The TSC can decide whether to defer the final analysis.

## 6.6 Actions arising from platform conclusions

The actions arising from platform conclusions will be specified in each DSA/DSSAP, but we provide some example actions below:

- Superiority: if a statistical decision of intervention superiority/inferiority (compared to another treatment) in a domain has occurred then, after review, a platform conclusion is declared and the less beneficial treatment may be removed from the domain. For a two-arm domain this will result in closure of the domain.
- Global superiority/optimal intervention: if an optimal intervention has been identified (according to the statistical decision criteria) then this may result in i) closure of the domain and a public disclosure of results (if the domain does not have a control, or control is incorporated into the calculation of the “best arm”), or ii) removal of the other experimental arms and randomisation may continue between the control and best intervention/experimental arm (if the calculation of the “best arm” did not include control and the domain has a control arm).
- Harm: if a statistical decision indicating harm for an intervention within a domain is reached, then, following review, a platform conclusion will be declared. The intervention’s allocation probability will be set to 0, and it will be removed from the set of available treatment options in that domain. For a two-arm domain this will result in closure of the domain.
- **Global inferiority/worst intervention: if a statistical decision indicating that an intervention is inferior compared to the other treatments within a domain is reached, then, following review, a platform conclusion will be declared. The intervention’s allocation probability will be set to 0, and it will be removed from the set of available treatment options in that domain.**
- Futility: some domains may use futility decision criteria to inform arm dropping or domain stopping (meaning that the intervention is unlikely to show a clinically important difference). In these instances, if a statistical decision of intervention futility in a domain has occurred, then, after review, a platform conclusion is declared and t**he intervention’s allocation probability will be set to 0 (and it will be removed from the set of available treatment options in that domain). In other situations the result may instead be communicated to the TSC and they will determine the appropriate** **action (public disclosure, removal of one intervention, no action) and no automatic adaptation may be triggered in this setting (non-binding futility).**
- **Equivalence: If a statistical decision indicating equivalence between two treatments within a domain is reached, the result will be communicated to the TSC, who will determine the appropriate action (e.g., public disclosure, removal of one intervention, or no action). No automatic adaptation is implemented in response to an equivalence decision (non-binding criteria).**
- Non-inferiority: if two treatments meet a prespecified non-inferiority trigger, the result will be communicated to the TSC and they will take the appropriate action (public disclosure, removal of one intervention, no action). There is no automatic adaptation when this trigger is met. The relevant DSAs will specify if this trigger is non-binding or binding.

Removal of interventions from two-arm domains result in suspension/closure of the domain.
A public disclosure of results would typically follow a domain closure, and future PROMPT participants (to whom the results are applicable) will likely be given the superior intervention from the domain (embedding) and randomised to treatments in the other domains.

RAR probability updates do not require approval from the DSMC.

# 7.0 Reporting

The analyses identified in this document will be included in future trial reports and manuscripts. Additional analyses may be specified in the DSSAPs as required. Exploratory analyses not necessarily identified here may be performed to augment the planned analyses. Any post-hoc or unplanned analyses not specified here (or in the DSSAPs) will be clearly identified in any statistical reports and manuscripts for publication.

A separate reporting template will be used for the interim analysis reporting (for the DSMC).

The study will be reported using the Consolidated Standards of Reporting Trials (CONSORT) guidelines (Schulz et al., 2010) and the extension for adaptive clinical trials (Dimairo et al., 2020). Findings will be made available through the PROMPT and other partner websites, as well as prepared for presentations at relevant scientific meetings.

## 7.1 Blinding

When publicly reporting the results of a platform conclusion, the number of participants allocated to each treatment arm in the domain, as well as the number who were ineligible or for whom the domain was unavailable, will be disclosed. If it is decided to publish results from an ongoing domain for treatment arms that have reached platform conclusions, then the results for the ongoing treatment arms (that have not reached a platform conclusion) will not be reported. To preserve blinding regarding the performance of treatment arms in other domains, information on allocation proportions for those domains will not be reported when presenting baseline characteristics for the reported domain.

## 7.2 Model Parameter Summaries

Where Bayesian statistical models have been used to inform inference, summaries of the model parameters and the pre-specified statistical quantities of interest for the domain will be reported.

For each model parameter we will report the posterior mean, median, standard deviation, and 95% credible intervals (CrIs; equal-tailed). Where a transformation is more appropriate, transformed parameters will be reported (e.g. odds ratios, etc.). Evidence of treatment effects will be expressed through posterior probabilities defined over the relevant parameter space (i.e. for relevant statistical quantities). In general, we will not report p-values (the exception being exploratory analyses) and will not adjust for multiplicity as analyses are undertaken in a Bayesian framework. (Appropriate threshold values will be chosen for statistical quantities to ensure sufficient control of error rates).

## 7.3 Participant Progression

CONSORT-style flow diagrams will be used to illustrate patient progression through the platform and domains. We will report number of patients screened who met inclusion criteria for the platform trial and the number excluded as they met one or more exclusion criteria. For each domain, we will then report the number of patients eligible for the domain, the number of participants enrolled in the domain, and the number randomised to each (unblinded) treatment arm. The CONSORT diagram will also detail the number of patients who were lost to follow-up, withdrew, and were included/excluded from the primary analysis. Reasons for ineligibility will be presented by platform, domain, and intervention (where applicable). We will report the number of patients who received their randomised allocation for the domain. Reasons for study withdrawal and treatment withdrawal will be presented by treatment group in each domain. If analytic strata are also employed, then these numbers will be presented for each domain/analytic strata combination.

## 7.4 Participant characteristics

Descriptive summaries of the baseline variables described below will be presented when reporting final analyses for each domain. Categorical variables will be presented using counts and percentages; continuous variables using the mean, median, standard deviation and interquartile range. Baseline characteristics will be presented in aggregate and by intervention, and will be reported separately for each domain/analytic strata combination.

Demographic:

- Age (years)
- Age group
- Sex
- ASA
- BMI
- Ethnicity
- Current place of residence (home, hospital/nursing facility)
- Planned ICU/HDU admission

Comorbidities / Medical Conditions:

- Hypertension
- Coronary artery disease
- Heart failure
- Myocardial Infarction
- Peripheral vascular disease
- Current smoker/vape
- Diabetes
- COPD
- Malignancy (presence of malignant tumour or cancer)
- Anaemia (pre-operative Hb <130g/L male and <120g/L female)
- Antibiotics within 7 days of surgery

Preoperative medications:

- Beta Blockers
- ACE inhibitor/ARB
- Aspirin
- Oral steroids
- Other Immunosuppressive drugs
- NSAID or COX-II inhibitor
- Oral antidiabetics
- Insulin
- GLP-1 agonists

Preoperative blood tests:

- Haemoglobin
- Creatinine
- White cell count

Intraoperative data:

- Surgical procedure performed
- Elective or expedited surgery

Other:

- EQ-5D-5L
- EQ VAS

We may also report domain specific baseline characteristics that will be specified in the DSAs.

## 7.5 Protocol adherence

Adherence and protocol deviations will be defined separately for each domain in the DSAs. Adherence to the treatment protocol and protocol deviations will be presented using descriptive statistics by treatment arm in each domain (and analytic strata; if applicable).

Site-level listings and summaries will be generated on a monthly basis to support ongoing monitoring of adherence to the treatment protocol. These reports will be used to identify, track, and manage protocol deviations in a timely manner.

## 7.6 Graphical and Descriptive Summaries

For each outcome, summaries will be presented in aggregate and by treatment group. Where longitudinal measures are available, cross-sectional summaries will be presented across time-points. Dichotomous outcomes will be summarised by counts and proportions in each category. Ordinal outcomes will be presented using stacked bar plots, cumulative probability plots, and will be summarised by the frequency of each outcome category. Time-to-event outcomes will be presented using Kaplan-Meier plots and summarised by percentiles of the Kaplan-Meier estimates where available.

## 7.7 Missing data

## The number and percentage of missing data will be reported for all baseline covariates and outcomes. When imputation is used, the variables with imputed values and the imputation method will be clearly documented.

Trial participants may discontinue their participation from the entire trial (based on the criteria outlined in the Master Protocol), or from individual domain-specific interventions. Therefore, participants may choose to withdraw from the entire platform (and thus any further data collection), or from continuing to receive an intervention within a specific domain while remaining enrolled in other domains within the platform and continuing follow-up. Where withdrawal occurs, the reasons will be documented in the participant CRF. All data collected up till the time of trial withdrawal will remain part of the study data set and included in analyses.

## 7.8 Changes to prespecified analyses

The unblinded PROMPT Analytic Team will monitor the primary model outputs, including numerical stability. Alternative models may be needed if there are unforeseen numerical, data, or modelling problems, and these adjustments to the model will be recorded and noted by the unblinded analytic team. Any alternatives (to the models outlined in the Current State Document) implemented by the PROMPT Analytic Team will be communicated to the DSMC and will be reported to the TSC in a way that does not risk unblinding of trial results.

The blinded statistical team will monitor scientific appropriateness of the statistical model and may receive blinded feedback from the unblinded analytic team regarding changes made to the model to improve the numerical stability. The blinded statistical team will maintain the Current State Document and update it as necessary.

# References

Angus DC, Berry S, Lewis RJ, et al. “The REMAP-CAP (Randomized Embedded

Multifactorial Adaptive Platform for Community-acquired Pneumonia) Study. Rationale and Design.” *Ann Am Thorac Soc.* 2020; **17**:879–91.

Berrios-Torres SI, Umscheid CA, Bratzler DW, et al. “Centers for Disease Control and Prevention Guideline for the Prevention of Surgical Site Infection”. *JAMA surgery.* 2017; **152**(8): 784-91.

Bofill Roig, M, Pavla K, Carl-Fredrik B, et al. “On Model-Based Time Trend Adjustments in Platform Trials with Non-Concurrent Controls.” *BMC Med Res Methodol. 2022;* **22:**228

Carpenter, B, Gelman A, Hoffman M, et al. “Stan: A Probabilistic Programming Language.” *Journal of Statistical* *Software.* 2016; **20**: 1–37.

Cellamare M, Ventz S, Baudin E, Mitnick CD, Trippa L. “A Bayesian response-adaptive trial in tuberculosis: the endTB trial.” *Clin Trials.* 2017;**14**:17–28.

Dimairo M, Pallmann P, Wason J. et al. “The adaptive designs CONSORT extension (ACE) statement: a checklist with explanation and elaboration guideline for reporting randomised trials that use an adaptive design.” *Trials.* 2020; **21**: 528.

European Medicines Agency. ICH E9 (R1) addendum on estimands and sensitivity analysis in clinical trials to the guideline on statistical principles for clinical trials. 2020. <https://www.ema.europa.eu/en/documents/scientific-guideline/ich-e9-r1-addendum-estimands-sensitivity-analysis-clinical-trials-guideline-statistical-principles_en.pdf>.

Fergusson D, Aaron SD, Guyatt G, Hébert P. Post-randomisation exclusions: the intention to treat principle and excluding patients from analysis. *BMJ.* 2002; **325(7365)**:652-4.

Fixed and Adaptive Clinical Trial Simulator (FACTS). 2025. Version 8.0. Austin (TX): Berry Consultants.

Mahar RK, McGlothlin A, Dymock M, et al. “A blueprint for a multi-disease, multi-domain Bayesian adaptive platform trial incorporating adult and paediatric subgroups: the Staphylococcus aureus Network Adaptive Platform trial. Trials”. 2023; **24**(1):795

R Core Team. “R: A language and environment for statistical computing, Vienna. Austria: R Foundation for Statistical Computing”. 2026.

Saville, BR., Berry DA, Berry NS, et al. “The Bayesian Time Machine: Accounting for Temporal Drift in Multi-Arm Platform Trials.” *Clinical* *Trials.* 2022; **19**(5): 490–501.

Schulz KF, Altman DG, Moher D; CONSORT Group. “CONSORT 2010 statement: updated guidelines for reporting parallel group randomised trials.” *BMJ*. 2010;**340**:c332.

StataCorp. “Stata Statistical Software: Release 17”. College Station, TX: StataCorp LLC; 2021.

Trippa L, Lee EQ, Wen PY, et al. “Bayesian adaptive randomized trial design for patients with recurrent glioblastoma.” *J. Clin. Oncol*. 2012; **30**:3258-63.

Viele K, Broglio K, McGlothlin A, Saville BR. “Comparison of methods for control allocation in multiple arm studies using response adaptive randomisation”. *Clin Trials*. 2020; **17**:52–60.

Wathen JK, Thall PF. “A simulation study of outcome adaptive randomization

in multi-arm clinical trials.” *Clin Trials.* 2017; **14**:432–40.

Zhao W. “Mass weighted urn design--A new randomization algorithm for unequal allocations.” *Contemp Clin Trials*. 2015; **43**:209-16.
